# Multi-Location Analysis of Cycling Forces and Impulses Across Posture, Cadence, and Gear Ratio

**DOI:** 10.3390/s26206559

**Published:** 2026-10-16

**Authors:** Philip X. Fuchs, Godwin Chinedu Uzomba, Meng-Yu Chen, Yin-Shin Lee, Tzyy-Yuang Shiang

**Affiliations:** 1 Department of Physical Education and Sports Sciences, National Taiwan Normal University, Taipei City 106, Taiwan; 81230005a@ntnu.edu.tw; 2 Department of Anatomy, Alex Ekwueme Federal University Ndufu-Alike, Abakaliki P.M.B. 1010, Nigeria; uzomba.godwin@funai.edu.ng; 3 Department of Sport and Kinesiology, National Taiwan Normal University, Taipei City 116, Taiwan; sweeetlee07@gmail.com

**Keywords:** bicycling, biomechanical phenomena, exercise therapy, ergonomics, rehabilitation

## Abstract

**Highlights:**

**What are the main findings?**

- Seated posture effects were concentrated at the handlebar, where the comfort configuration reduced vertical peak force, cycle-averaged force, and impulse.
- Increasing cadence reduced impulse at all contact points and increased pedal-force magnitude, whereas heavy gear increased pedal loading while reducing saddle and handlebar loading.

**What are the implications of the main findings?**

- Multi-contact assessment is valuable for region-specific load management because cycling adjustments can modify loading at specific rider–bicycle interfaces without detectable changes at others.
- Adjustment choices should depend on the mechanical target: cadence is particularly relevant to force-time characteristics, whereas gear ratio is more relevant to vertical load redistribution across rider–bicycle contacts.

**Abstract:**

This study aimed to investigate differences in forces and impulses at five rider–bicycle contacts (bilateral grips, saddle, and bilateral pedals) across posture, cadence, and gear ratio. In a randomized, repeated-measures laboratory design, 10 male and 10 female recreational cyclists completed 18 combinations of three seated postures, three cadences, and two gear ratios on a stationary indoor bicycle sensing system (50 Hz). Three-way repeated-measures ANOVAs with Holm correction examined body-mass-normalized vertical peak force, cycle-averaged force, and impulse per cycle at *p* < 0.05. Posture affected all three outcomes at both grips (*p* < 0.01, ηp^2^ = 0.360–0.552) but not the saddle or pedals (*p* = 0.821–1.000, ηp^2^ = 0.027–0.157). Cadence affected grip and saddle impulse and all pedal outcomes (*p* < 0.01, ηp^2^ = 0.544–0.908); higher cadence reduced impulse at all contacts and increased pedal-force magnitude. Heavy gear increased all pedal outcomes and reduced all grip and saddle outcomes compared with light gear (*p* < 0.001, ηp^2^ = 0.593–0.981). Interactions were limited, and sensitivity analyses reproduced all main-effect conclusions. Posture effects were concentrated at the handlebar, cadence primarily modified force-time characteristics and pedal-force magnitude, and gear ratio redistributed vertical loading between the pedals and other contacts. These findings support a purpose-dependent framework for region-specific mechanical load management in comparable indoor cycling conditions.

## 1. Introduction

Cycling is widely used in health-related exercise and rehabilitation [1]. During cycling, repetitive lower-limb motion generates pedal forces, crank torque, and external power output that propel the bicycle forward [2,3]. Accordingly, pedal forces are central to cycling performance because they determine propulsion effectiveness and reflect low-er-extremity contributions throughout the pedal cycle [2,3]. In typical seated cycling, the vertical force component constitutes a major component of pedal loading and propulsion, particularly in recreational cyclists [3,4]. Vertical forces are therefore relevant to lower-extremity mechanical loading, joint stress, and rehabilitation [5,6].

The rider interacts with the bicycle through multiple contact points, including the pedals, saddle, and handlebar; forces at these interfaces contribute to posture control, stability, and body-weight transmission within the rider–bicycle system [2,7]. From a medical and applied perspective, this broader view is important because cycling-related load is not restricted to the lower limbs. The saddle and handlebar expose other body regions to repeated mechanical demands, including the pelvis, perineal region, trunk, shoulders, arms, and hands [8,9,10]. The handlebar interface is especially relevant because prolonged pressure at the hand-handlebar contact area has been linked to ulnar nerve compression and cyclist’s palsy [8]. In rehabilitation, bicycle fitting, and symptom-oriented exercise prescription, a relevant question is therefore how loads are distributed across rider–bicycle contacts. A change that has little effect on pedal force may still alter support demands at the saddle or handlebar, which is relevant when the goal is to reduce local loading or avoid aggravating symptoms and discomfort in specific body regions [2,9,10]. Comfort is important because tolerable and enjoyable exercise can support adherence and longer-term integration of physical activity into daily life [11,12]. This broader perspective is also relevant to bicycle fitting, in which rider–bicycle contacts are adjusted to improve comfort, reduce pain, and maintain a sustainable riding posture [13,14].

Among the factors that may alter this distribution, riding posture is especially relevant [2,13]. Changes in posture modify trunk inclination and the rider’s overall body configuration and may thereby alter support demands among the upper body, pelvis, and lower limbs [2,15]. Previous research on cycling posture has often focused on performance-oriented questions and on comparisons between fundamentally different posture types, such as seated versus standing cycling or more upright seated riding versus the markedly forward-inclined racing position used to reduce aerodynamic drag and optimize competitive performance [2,7,13]. These comparisons are valuable, but they do not fully address the more moderate variations within the broader category of upright seated posture that are common in recreational cycling, bicycle fitting, and therapeutic exercise. In these settings, the most relevant posture is usually not a racing configuration but a comfort-oriented seated posture that prioritizes tolerable loading, comfort, and symptom management over maximal aerodynamic efficiency [9,13,15]. Bicycle fitting commonly involves relatively small adjustments within this seated posture type rather than shifts to fundamentally different riding techniques [13,14,15]. Yet, the effects of variations among practically relevant seated bicycle-fit configurations on vertical peak force, cycle-averaged force, and impulse per cycle across the handlebar grips, saddle, and pedals remain insufficiently understood [2,13].

Cadence and gear ratio are likewise important because they influence cycling mechanics differently [4,5,16]. Gear ratio changes the external force requirements of the task more directly, whereas cadence changes movement velocity, cycle timing, and neuromuscular demands during each pedal cycle [4,5,16]. Both variables are relevant to performance and health. Cadence and workload-related variables affect pedal-force application, joint-specific power, knee joint loading [4,5,16], and rehabilitation outcomes [1,17]. In training and therapy, cadence and gear ratio are often manipulated to achieve different aims, such as increasing or reducing exercise intensity, changing neuromuscular demand, improving movement tolerance, or limiting local mechanical stress [1,16,17]. For this reason, understanding their effects on force production and force distribution is relevant not only to performance optimization but also to clinically informed exercise prescription.

In practice, posture, cadence, and gear ratio are often modified together to improve comfort, reduce symptoms, maintain training quality, or optimize performance [13,14,17]. Nevertheless, research has tended to focus primarily on pedal forces, for which dedicated measurement systems are well established [18]. As a result, much less is known about the combined effects of these variables on forces and impulses at other clinically and practically relevant contacts, such as the saddle and handlebar grips [2,10,13]. Another limitation is that studies examining pedaling strategy and cycling mechanics have focused mainly on male cyclists, with female cyclists comparatively underrepresented [13].

The primary purpose of this study was to investigate differences in vertical peak force, cycle-averaged force, and impulse per cycle at the handlebar grips, saddle, and pedals across different postures, cadences, and gear ratios. A secondary purpose was to examine potential interaction effects. It was hypothesized that an athletic, more forward-inclined configuration would increase handlebar loading, higher cadence would reduce impulse per cycle across all contacts, and heavy gear ratio would increase pedal loading.

## 2. Materials and Methods

### 2.1. Participants

Twenty recreational cyclists [10 males: age 26.0 ± 7.0 (20–44) years, height 170.5 ± 5.4 (159–180) cm, body mass 69.98 ± 10.63 (53.6–92.7) kg; 10 females: age 29.0 ± 14.2 (20–62) years, height 161.9 ± 4.7 (156–170) cm, body mass 52.83 ± 5.37 (46.8–61.3) kg] participated in this study. An inclusion criterion was routine training at high cadences (i.e., ≥100 rpm) [19]. The participants’ preferred cadences were 69 ± 9 rpm among females and 82 ± 6 rpm among males, comparable to previously reported values [19]. Cycling experience and weekly training volume were not documented. Volunteers were excluded if they were not in good health or experienced lower extremity or neurological injuries within six months before data collection.

A design-based power analysis was conducted in R version 4.6.0 (R Core Team, Vienna, Austria) using the Superpower package version 0.2.4.1 [20]. Power was calculated analytically for each primary main effect in a 3 (posture) × 3 (cadence) × 2 (gear ratio) repeated-measures design, assuming a moderate effect size (f = 0.25), a standardized condition-level SD of 1, nominal α = 0.05, and compound-symmetric within-participant correlations of r = 0.50 across the 18 repeated conditions. The required sample size at 80% power was 6 participants for each main effect. The analysis was designed to assess sensitivity for the primary main effects and did not establish 80% power for every Holm-adjusted outcome or for secondary interaction effects. Complete code for reproducing the power analysis is provided in Supplementary Code S1.

The study was approved by an institutional ethics committee (approval number: 202205HM064) in accordance with the Declaration of Helsinki, and all participants provided written informed consent after the experimental procedures had been explained.

### 2.2. Measures

The participants’ body mass was obtained via a Super-View HW-3030 scale (Kong Ho Instruments Co., Ltd., New Taipei City, Taiwan).

A commercial indoor bicycle system (Dynamic Cycling Fit DCF Bike Pro, Giant Manufacturing Co., Ltd., Taichung, Taiwan) measured vertical forces during cycling via five strain-gauge full-bridge sensors (50 Hz). Comparable cycling-force research has used a validated high-precision power-meter system operating at 50 Hz to quantify dynamic force profiles [21]. Sensors were located at the saddle base underside, both lateral handlebar grip sections adjacent to the brake lever clamps, and both crank-spindle interfaces. The system uses the same strain-gauge full-bridge sensor type and acquisition architecture at all five locations. Signals from all five force channels were acquired simultaneously. The system incorporated a differential amplifier and a 24-bit analog-to-digital converter and performed automated calibration during system startup. Strain-gauge-based force sensing is commonly used in applied cycling research to quantify cyclist–bicycle interface forces at the pedals, handlebar grips, and saddle [2,22,23]. Development, validation, and reproducibility studies of other cycling-specific strain-gauge measurement systems have reported low calibration errors, adequate dynamic response across cycling-relevant frequencies, and high test–retest reproducibility [24,25,26,27,28]. These studies characterize comparable measurement approaches but do not constitute device-specific validation of the present system.

As an independent verification of the present system, a standard 10 kg weight was placed on the saddle 10 times. The resulting measurements showed a mean difference from the reference load of 0.34 ± 0.28 kg, a relative root mean square error of 4.3%, 95% limits of agreement of −2.2 to 9.0%, and an intra-session coefficient of variation of 2.4%. The system was leveled before measurement according to the manufacturer’s installation procedure.

### 2.3. Design and Procedures

This study used a randomized, repeated-measures laboratory design. Each participant attended a setup session followed by a single experimental session. During the setup session, the participant-specific bicycle setup, three posture conditions, and self-selected cadence were established. Participants cycled in all three postures, with familiarization individualized rather than standardized by duration. Participants were allowed to continue cycling until they felt familiar and comfortable with the bicycle and experimental setup. During the experimental session, each participant completed 18 cycling trials, combining three riding postures, three cadences, and two gear ratios in randomized order. Vertical peak force, cycle-averaged vertical force, and vertical impulse per cycle were compared within participants across conditions at the handlebar grips, saddle, and pedals.

Participants cycled on a stationary indoor cycle ergometer (Dynamic Cycling Fit DCF Bike Pro, Giant Manufacturing Co., Ltd., Taichung, Taiwan) while wearing clip-in shoes. They performed stable pedaling at three cadences [self-selected, slow (i.e., self-selected − 10 rpm), and quick (i.e., self-selected + 10 rpm)] and two gear ratios (heavy: 50/15, light: 34/15) in three distinct bicycle configurations on a training stand, with trial order randomized. Gear ratio was predefined by the system setup, and cadence was monitored throughout the trials in real time at 200 Hz via an inertial measurement unit (IMU) at the crank (Trigno Avanti, Delsys, Natick, MA, USA). External resistance, power output, crank torque, and work rate were not recorded. A trial was completed when the participant performed 15 consecutive pedaling cycles at a stable cadence (i.e., within ±3 rpm of the target cadence). Trial duration was therefore not fixed and was not separately recorded. No fixed rest interval was prescribed; participants were allowed to rest between trials as needed. No participant requested additional rest beyond the time required to adjust the bicycle or experimental condition between trials. Fatigue and carryover effects were not formally assessed; randomized trial order, short low-intensity trials, and rest as needed minimized these effects.

**Figure 1:**
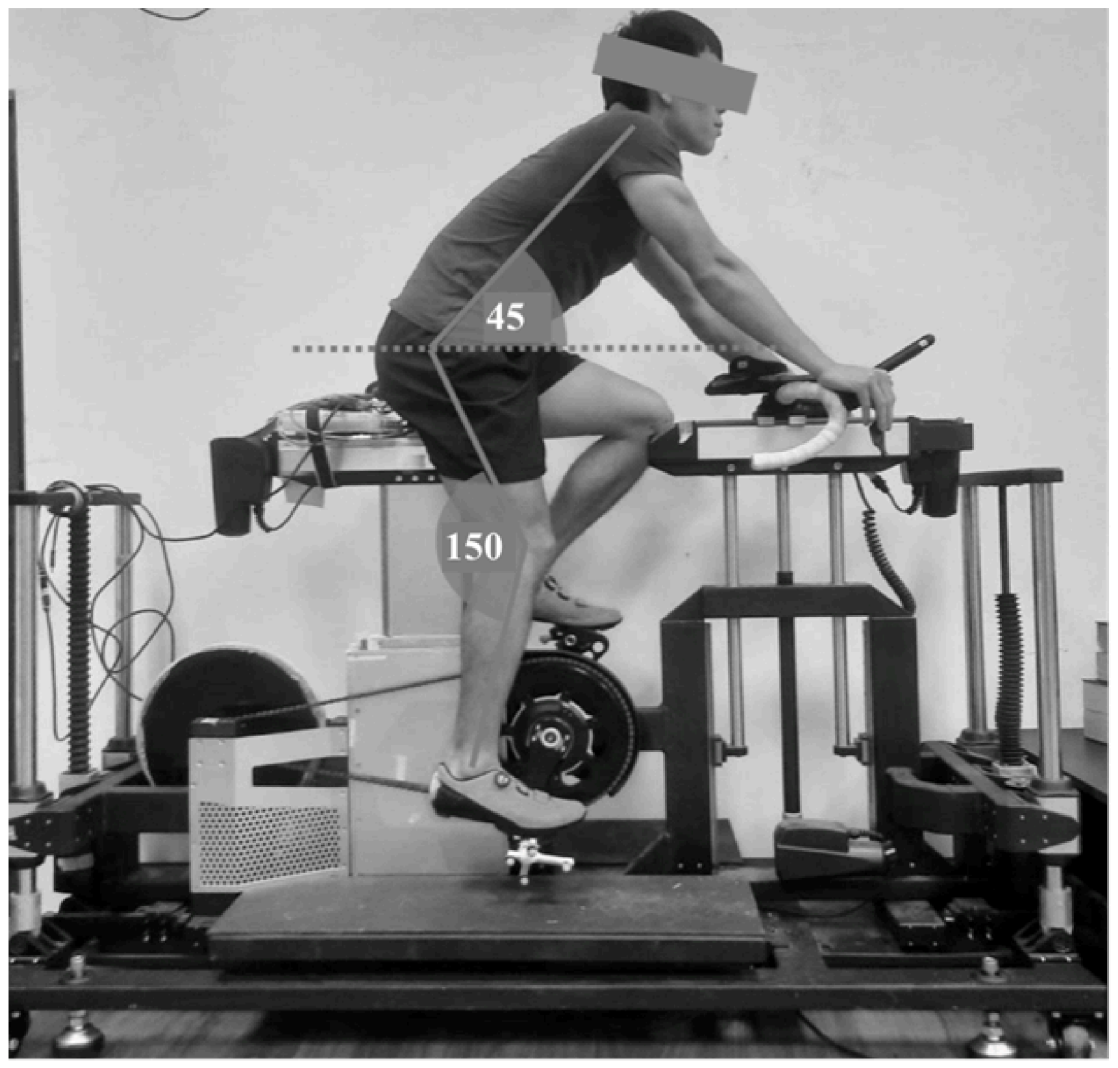
Bicycle system and setup for the neutral posture.

The neutral posture was achieved through saddle and handlebar adjustments that produced a 150° knee angle with the pedal at its lowest point and a 45° trunk angle relative to the horizontal plane (see Figure 1) [29]. Saddle height was normalized to greater trochanter height [30] and maintained across conditions. A long steel ruler, a protractor, and a level were used to obtain knee and trunk angles. For the comfort posture, the handlebar position was set 5 cm higher than in the neutral posture to create a more upright, comfort-oriented riding position. For the athletic posture, the handlebar position was set 5 cm lower and the saddle was moved 5 cm forward compared with the neutral posture to create a more forward-inclined, performance-oriented riding position. These standardized adjustments represented practically relevant posture differences, consistent with literature indicating that higher handlebars are associated with more upright, comfort-oriented positions [31], whereas lower handlebars and forward saddle positions contribute to more aggressive postures through altered trunk and hip angles [13]. The three posture conditions represented standardized bicycle-fit configurations rather than isolated manipulations of individual bicycle-fit parameters. Resulting trunk, hip, and knee angles in the athletic and comfort postures, as well as the resulting effective seat-tube angle in the athletic posture, were not recorded.

The study followed guidelines for reporting bicycle setup dimensions [32], and the eight consensus items were summarized below:

- Bicycle or cycle ergometer: Ergometer
- Tools for measurement of bicycle dimensions: Steel ruler, protractor, and level
- Saddle height: Individually adjusted during the neutral setup and maintained across conditions
- Saddle setback: Individually established for the neutral and comfort conditions and moved 5 cm forward for the athletic condition
- Effective seat tube angle for the neutral setup: 74 degrees
- Crank length: 170 mm
- Vertical difference between handlebar and saddle height: Condition-specific as described above
- Diagonal difference between handlebars and saddle height: Condition-specific as described above

Force data were processed via Matlab 2020b (The MathWorks, Inc., Natick, MA, USA) with a 10 Hz fourth-order zero-phase Butterworth low-pass filter [33,34] and trimmed to 15 consecutive pedaling cycles at a stable cadence. Cycles were identified using the crank IMU from one top-pedal position to the next, and vertical impulse was integrated over each complete cycle at all five measurement locations. Peak vertical force (N/kg), cycle-averaged vertical force (N/kg), and vertical impulse per cycle (N·s/kg) at all five contacts were normalized to body mass, and the average across the 15 cycles was used for further analysis.

### 2.4. Statistical Analysis

Statistical analyses were performed in R version 4.6.0 (R Core Team, Vienna, Austria). All records were screened for duplicated recordings, extreme outliers (mean ± 3SD), and implausible negative or zero values. Four participants were excluded from pedal analyses because of duplicated left- and right-pedal recordings, suggesting preprocessing or export errors. One additional participant was excluded from the left-pedal impulse analysis because of missing data and from the right-pedal impulse analysis because of an implausible negative value. Consequently, sample sizes were *n* = 20 for handlebar and saddle outcomes, *n* = 16 for pedal peak-force outcomes, and *n* = 15 for pedal impulse and cycle-averaged force outcomes.

Normality of participant-level within-subject contrast scores was assessed via Q–Q plots and Shapiro–Wilk tests. The primary analysis used the original measurement scale. As deviations from normality occurred in 14.9% of contrast components, all factorial analyses and corresponding follow-up comparisons were repeated after natural-log transformation as a sensitivity analysis. Sphericity was assessed using Mauchly’s test. When sphericity was violated, Greenhouse–Geisser corrections were applied for ε < 0.75 and Huynh-Feldt corrections for ε ≥ 0.75.

For each outcome, a 3 (posture: comfort, neutral, athletic) × 3 (cadence: slow, self-selected, quick) × 2 (gear ratio: light, heavy) repeated-measures ANOVA was performed. Effect sizes were reported as partial eta squared (ηp^2^) without applying generic benchmarks developed for different fields [35]. Sex was not included as an inferential factor because the study was not designed to test sex differences or equivalence. Sex-stratified means and standard deviations were presented descriptively.

To address multiplicity across outcomes, Holm correction was applied across the 15 outcome variables separately for each factorial effect. Significant three-level main effects were followed by Holm-adjusted paired comparisons. Significant cadence × gear-ratio interactions were explored using simple effects of cadence within each gear-ratio level and of gear ratio within each cadence level, with Holm correction applied within each follow-up family. Statistical significance was set a priori at *p* < 0.05.

**Figure 2:**
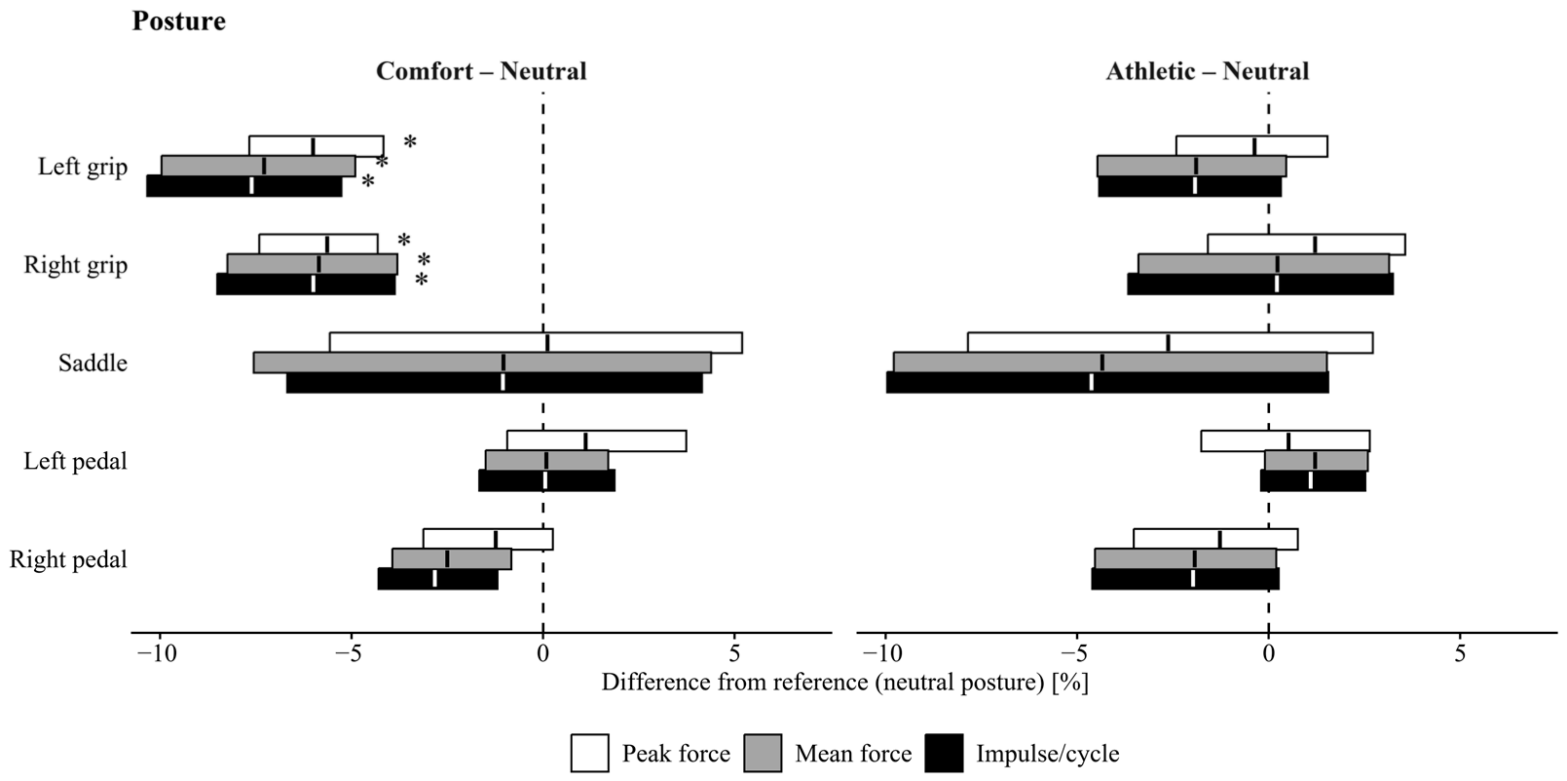
Relative differences in vertical peak force, vertical cycle-averaged (mean) force, and vertical impulse per cycle across riding postures at five rider–bicycle contacts. Horizontal bars indicate unadjusted 95% confidence intervals; vertical ticks indicate point estimates; * indicates significant contrasts after Holm correction. Each data panel represents the indicated contrast relative to the neutral posture. Positive values indicate higher values in the comfort or athletic posture than in the neutral posture, whereas negative values indicate lower values.

**Figure 3:**
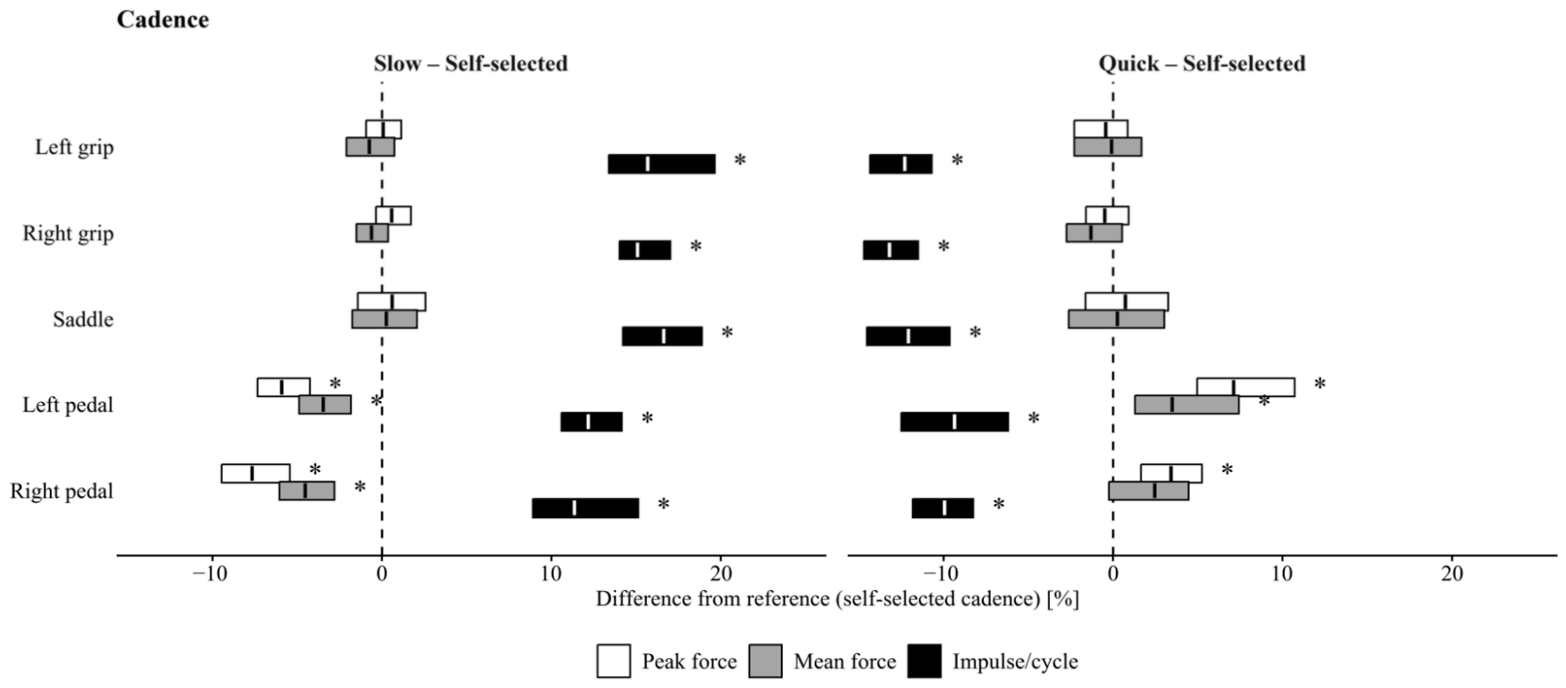
Relative differences in vertical peak force, vertical cycle-averaged (mean) force, and vertical impulse per cycle across cadences at five rider–bicycle contacts. Horizontal bars indicate unadjusted 95% confidence intervals; vertical ticks indicate point estimates; * indicates significant contrasts after Holm correction. Each data panel represents the indicated contrast relative to the self-selected cadence. Positive values indicate higher values at the slow or quick cadence than at the self-selected cadence, whereas negative values indicate lower values.

**Figure 4:**
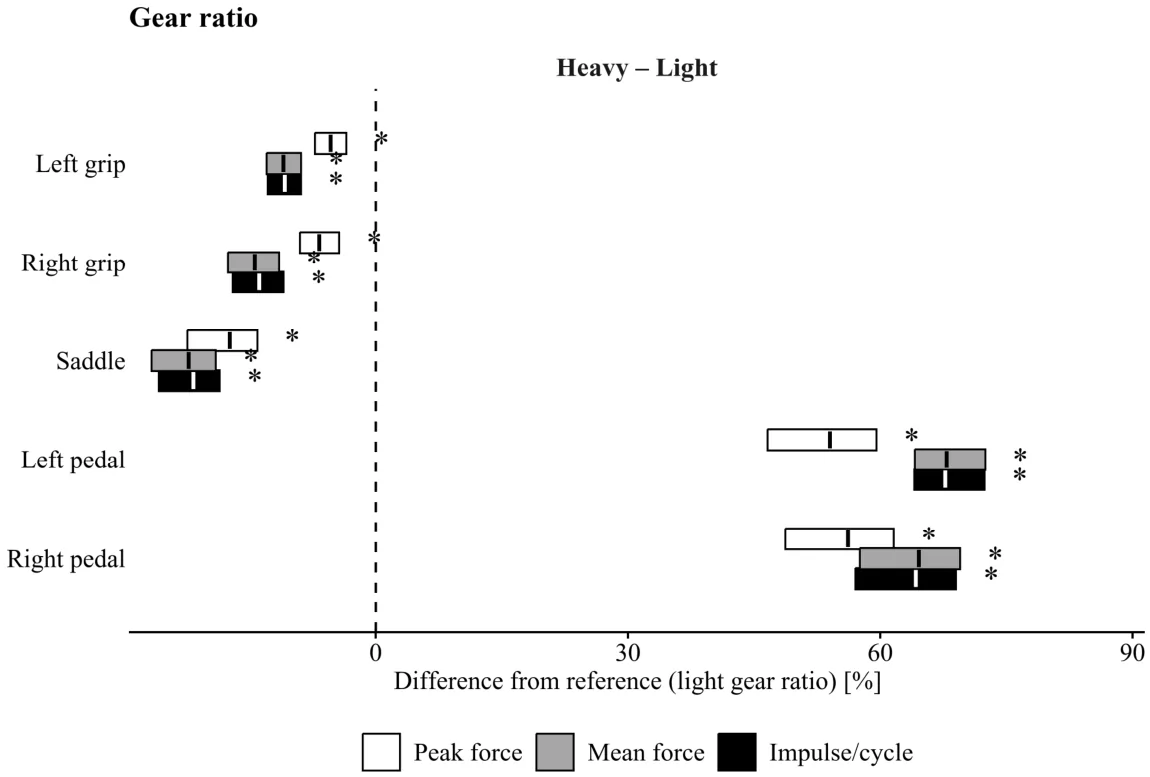
Relative differences in vertical peak force, vertical cycle-averaged (mean) force, and vertical impulse per cycle across gear ratios at five rider–bicycle contacts. Horizontal bars indicate unadjusted 95% confidence intervals; vertical ticks indicate point estimates; * indicates significant contrasts after Holm correction. All plotted values represent the heavy-minus-light contrast. Positive values indicate higher values under the heavy-gear condition, whereas negative values indicate lower values.

Figure 2, Figure 3 and Figure 4 were generated in R using the boot and ggplot2 packages. For each contrast, participant-level marginal means were calculated by averaging across the non-focal experimental factors, and relative differences were expressed as percentages of the reference-condition mean. Bias-corrected and accelerated (BCa) 95% confidence intervals were obtained using paired participant-level bootstrap resampling with 20,000 resamples.

### 2.5. Use of Generative Artificial Intelligence Tools

ChatGPT (OpenAI; GPT-5.4 and GPT-5.6 Sol) was used to support supplementary literature exploration following the authors’ conventional literature searches, development and verification of R code for statistical analyses and visualization, checks of result interpretation, and text revision. Elsevier LeapSpace™ (https://researcher.elsevier.com/cny/ (accessed on 13 October 2026)) was used for supplementary literature exploration and claim-level evidence checking. Neither tool independently selected literature for inclusion in the manuscript, conducted the final statistical analyses, or determined the interpretation of the results. All identified literature was checked against the original publications, statistical code and outputs were reviewed by the authors, and all AI-assisted suggestions and manuscript revisions were critically evaluated and approved by the authors.

## 3. Results

Figure 2, Figure 3 and Figure 4 displayed relative differences in forces and impulses across posture, cadence, and gear ratio at all five measurement locations. Supplementary Tables S1–S4 provided sex-stratified descriptive data as means ± standard deviations.

Posture affected all three outcomes at both handlebar grips (all *p* < 0.01, ηp^2^ = 0.360–0.552) but not pedal or saddle outcomes (*p* = 0.821–1.000, ηp^2^ = 0.027–0.157). Post hoc comparisons showed lower handlebar peak force, impulse, and cycle-averaged force in the comfort posture than in the neutral and athletic postures (all *p* < 0.01, ηp^2^ = 0.406–0.745). Neutral and athletic postures did not differ (*p* = 0.133–0.902, ηp^2^ = 0.001–0.115).

Cadence affected handlebar and saddle impulse, pedal peak force and impulse, and pedal cycle-averaged force (all *p* < 0.01, ηp^2^ = 0.544–0.908), but not handlebar or saddle peak force or cycle-averaged force (*p* = 1.000, ηp^2^ = 0.001–0.078). Increasing cadence progressively reduced impulse at all locations and increased pedal peak force (all *p* < 0.01, ηp^2^ = 0.487–0.917). Cycle-averaged pedal force also increased with cadence; five of six pairwise comparisons were significant (*p* < 0.05, ηp^2^ = 0.273–0.717), while self-selected and quick cadence did not differ for the right pedal (*p* = 0.060, ηp^2^ = 0.230).

Heavy gear increased all three pedal outcomes but reduced all three handlebar and saddle outcomes compared with light gear (all *p* < 0.001, ηp^2^ = 0.593–0.981).

None of the posture × cadence, posture × gear-ratio, or posture × cadence × gear-ratio interactions was significant (*p* = 0.159–1.000, ηp^2^ = 0.001–0.169). Cadence × gear-ratio interactions were significant for left- and right-pedal impulse and right-grip cycle-averaged force (*p* = 0.002–0.003, ηp^2^ = 0.364–0.472), but not for the other outcomes (*p* = 0.185–1.000, ηp^2^ = 0.026–0.245). Both pedal impulses decreased progressively with increasing cadence within both gear-ratio levels (all *p* < 0.05, ηp^2^ = 0.344–0.886), while heavy gear produced greater impulse than under light gear at each cadence (all *p* < 0.001, ηp^2^ = 0.932–0.961). For right-grip cycle-averaged force, slow cadence produced lower values than self-selected and quick cadences under light gear but higher values under heavy gear (*p* = 0.011–0.046, ηp^2^ = 0.272–0.410). Self-selected and quick cadences did not differ under either gear ratio (*p* = 0.277–0.615, ηp^2^ = 0.014–0.112), and heavy gear produced lower cycle-averaged force than under light gear at each cadence (all *p* < 0.001, ηp^2^ = 0.536–0.782).

Log-transformed sensitivity analyses reproduced all main-effect conclusions. All post hoc conclusions for significant main effects were also reproduced except the left-pedal comparison between self-selected and quick cadence, which became nonsignificant after transformation (*p* = 0.057, ηp^2^ = 0.235). The cadence × gear-ratio interactions for left- and right-pedal impulse became nonsignificant after transformation (*p* = 1.000, ηp^2^ = 0.014 and 0.101, respectively), whereas the interaction for right-grip cycle-averaged force remained significant and the interaction for right-grip impulse became significant (both *p* < 0.01, ηp^2^ = 0.369). Right-grip impulse decreased with increasing cadence within both gear-ratio levels (all *p* < 0.001, ηp^2^ = 0.811–0.962) and was lower under heavy than under light gear at each cadence (all *p* < 0.001, ηp^2^ = 0.505–0.819).

Supplementary Tables S5–S13 provided complete F statistics, degrees of freedom, exact unadjusted and Holm-adjusted *p* values, and partial η^2^ with 95% confidence intervals for all conducted tests.

## 4. Discussion

### 4.1. Main Findings

This study examined the effects of riding posture, cadence, and gear ratio on vertical peak force, cycle-averaged force, and impulse per cycle at the handlebar, saddle, and pedals. Posture effects were concentrated at the handlebar. Cadence distinguished force magnitude from force-time accumulation within each cycle. Gear ratio redistributed vertical loading between the pedals and the handlebar and saddle. These interpretations are consistent with literature showing posture-related handle forces and broader biomechanical effects of positional adjustments [2,13], as well as cadence- and workload-related effects on cycling force application and lower-limb loading [4,5,16,19].

### 4.2. Posture, Cadence, and Gear-Ratio Effects

Posture effects were concentrated at the handlebar, with lower peak force, cycle-averaged force, and impulse in the comfort configuration than in the neutral and athletic configurations. This pattern is biomechanically plausible because changes in bicycle configuration can alter trunk, pelvis, shoulder, and elbow kinematics and upper-limb muscle activation [13,15]. Extending previous evidence focused largely on more substantial posture changes such as seated-to-standing cycling [2,13], the present findings show that moderate seated bicycle-fit adjustments can also alter handlebar loading without detectable changes in vertical saddle or pedal loading.

Cadence produced a distinct force-time pattern: impulse per cycle decreased at higher cadences, partly because shorter cycle duration reduces integration time. This was particularly clear at the handlebar and saddle, where peak and cycle-averaged forces remained mostly unchanged while impulse decreased, consistent with previous findings [36]. At the pedals, higher peak and cycle-averaged vertical forces occurred alongside lower impulse, suggesting changes in both force magnitude and temporal structure. Accordingly, the lower impulse per cycle at higher cadence should not be interpreted as lower accumulated loading over a fixed riding duration. Although constant-power research reported lower peak normal pedal forces with increasing cadence [4], vertical pedal force also contains gravity- and inertia-related components that increase with cadence [3]. Thus, the greater vertical pedal forces observed at higher cadence may partly reflect inertial contributions and should not prematurely be interpreted as greater muscular or propulsive force.

Gear ratio produced a coherent multi-contact pattern: heavy gear increased pedal forces and impulses while reducing values at the saddle and handlebar, consistent with a shift in vertical loading toward the pedals. Previous research has established greater pedal loading with increasing mechanical demand [4,5], gear-ratio effects on pedal-force effectiveness [37], and coupling between greater pedal loading and reduced saddle support as power increases [38]. The present findings extend this pattern to reduced vertical handlebar loading under heavy gear. Interpretation should remain specific to vertical downward force because greater crank power can increase three-dimensional upper-limb kinetics, including handlebar pulling [39]. Gear ratio appears to modify how vertical mechanical loading is distributed across rider–bicycle contacts rather than uniformly affecting loading throughout the system.

### 4.3. Interactions and Sex-Stratified Descriptive Findings

The main-effect patterns appeared generally consistent across the tested conditions, although interaction evidence was limited. The few significant cadence × gear-ratio interactions did not form a coherent pattern across contact points or outcomes and were mostly not robust to log transformation. Previous cycling studies reporting interactions have generally examined broader cadence and workload ranges [36,40] or more pronounced posture differences [41], which may accentuate interactions.

Sex-stratified descriptive data indicated that the principal directional patterns were present in both females and males. The comfort configuration generally reduced handlebar loading, heavy gear increased pedal loading while reducing saddle and handlebar loading, and increasing cadence increased pedal peak force while reducing impulse per cycle. The recurrence of these patterns suggests that the pooled effects were most likely not driven by one sex. Previous cycling research has reported similar condition-related responses [42]. Females showed numerically greater pedal impulses and saddle forces and impulses, which may partly reflect their lower absolute cadences and longer cycle durations [36,43]. These observations were not tested inferentially.

### 4.4. Practical Implications

The present findings suggest a provisional, purpose-dependent framework of cycling adjustments. For modifying vertical handlebar support demand, bicycle configuration appears to be the most direct lever; the comfort configuration reduced handlebar loading without detectable changes at the saddle or pedals. Cadence is more relevant to force-time characteristics; since higher cadence reduced impulse per cycle while increasing vertical pedal-force magnitude, cadence selection should reflect the primary mechanical target (per-cycle force accumulation or force magnitude). Importantly, lower impulse per cycle does not necessarily indicate lower accumulated loading over a ride of the same duration, because more cycles occur at higher cadence and cycle-averaged pedal force increased. Gear ratio appears most useful for redistributing vertical loading across rider–bicycle contacts, with heavy gear shifting loading toward the pedals, away from the saddle and handlebar. Within the tested conditions, these effect patterns appeared mostly consistent across factor combinations. Therefore, this framework may cautiously inform interpretation of individual-factor adjustments under comparable conditions.

For clinicians and therapists, these findings provide a broader mechanical basis for tailoring cycling exercise because cadence, workload, and bicycle configuration can modify lower-limb and joint loading in rehabilitation populations [5,44,45,46]. When upper-limb symptoms are relevant, bicycle fitters and clinicians may target handlebar height because repeated hand–handlebar loading has been associated with median and ulnar neuropathies [8,47]. Bicycle fitters may extend dynamic, workload-specific assessment approaches [48] by incorporating multi-contact kinetics to detect load shifts. Although bicycle fitting has been associated with pain and comfort outcomes [14], the additional clinical benefit of such kinetic information remains unclear. For bicycle-fitting system developers, simultaneous sensing across contacts may help identify load shifts not captured by assessments limited to pedal kinetics or bicycle geometry. Sports scientists may use multi-contact measurements to characterize effects of cycling interventions across the rider–bicycle system beyond propulsion, particularly when non-pedal contact mechanics are relevant.

Future multi-contact cycling assessment could integrate kinetic measurements with complementary data using intelligent computational methods. Recurrent neural networks have predicted three-dimensional pedal forces and lower-limb joint kinetics from cycling kinematics, demonstrating the feasibility of computationally deriving kinetic information from motion data [49]. Recent musculoskeletal studies have combined image-derived and medical information for explainable automated diagnosis [50], fused complementary imaging modalities for diagnostic support [51], and automated multi-tissue segmentation [52]. These principles could inform future integration of multi-contact forces with kinematic and clinical data for individualized cycling assessment.

### 4.5. Limitations

Several limitations should be considered. First, the study quantified only vertical peak force, cycle-averaged vertical force, and vertical impulse per cycle rather than all directional components. These variables are relevant to mechanical loading and are practically interpretable [3,4], but they do not capture power or isolate the crank-perpendicular component that determines crank torque [3]. External resistance, power output, torque, and work rate were not recorded. Accordingly, the cadence and gear-ratio effects characterize the tested conditions rather than effects isolated under quantified or matched external mechanical demand. Second, the tests were performed on an indoor training stand, which improves experimental control but may not represent outdoor cycling, where steering demands, balance perturbations, terrain, and vibration can alter load sharing across contacts [13,23]. Third, the sample consisted of physically active recreational cyclists familiar with high cadences and clip-in shoes. The findings are therefore most applicable to recreational and physically active populations familiar with cycling exercise, and caution is warranted when extrapolating them to different groups, such as novices or patients with substantially reduced physical capacity. Fourth, the independent verification of the force-measurement system was limited to repeated static loading at the saddle. Device-specific linearity, hysteresis, cross-talk, and dynamic response characteristics across all measurement locations remain unknown. Fifth, the posture conditions represented composite bicycle-fit configurations involving coordinated changes in handlebar and saddle position rather than isolated changes in body positioning. Sixth, fatigue and carryover effects were not formally assessed, although randomized trial order, short low-intensity trials, and rest as needed minimized these effects. Finally, although females and males were included, the study was not designed to test sex differences or equivalence. Sex-stratified results were presented descriptively, but no conclusions regarding sex differences or similarity were drawn.

## 5. Conclusions

Riding posture, cadence, and gear ratio influenced force and impulse at rider–bicycle contacts during the tested indoor cycling configurations, but their effects were not equally distributed across locations or outcomes. Posture effects were concentrated at the handlebar, whereas cadence primarily affected force-time characteristics and pedal-force magnitude, and gear ratio produced a consistent redistribution of vertical loading across contact points. These findings suggest a provisional practical framework of adjustment levers: posture appears to be a more direct tool for modifying upper-limb support demand, cadence for modifying force-time characteristics, and gear ratio for redistributing vertical loading between the pedals and other contacts. This information may support load management in rehabilitation, recreational cycling, and bicycle fitting, where the aim is to address region-specific mechanical demands across multiple rider–bicycle contacts. Future research may extend this approach through full three-dimensional force measurements, direct assessments of comfort, symptoms, and physiological responses, larger samples to test sex differences, and evaluations of outdoor or field-based conditions.

## Data Availability

The raw data supporting the conclusions of this article will be made available by the authors on request.

## Supplementary Materials

The following supporting information can be downloaded at: https://www.mdpi.com/article/10.3390/s26206559/s1, Code S1: Power analysis code; Tables S1–S4: Descriptive results; Tables S5–S13: Inferential results.
