# Supplementary material for: Multi-Location Analysis of Cycling Forces and Impulses Across Posture, Cadence, and Gear Ratio
Source: Sensors (Basel). 2026 Oct 16;26(20):6559. doi: 10.3390/s26206559 (PMC13708784; doi:10.3390/s26206559)
Supplement: Supplementary file 1 [file sensors-26-06559-s001.zip › Code S1 Power analysis code.pdf]

```r
# ======================================================================
# Supplementary Code S1
# Design-based power analysis for the primary main effects
#
# Experimental design:
#   3 posture levels x 3 cadence levels x 2 gear-ratio levels
#   All factors repeated within participants
#
# Power-analysis assumptions:
#   Cohen's f = 0.25
#   Nominal alpha = 0.05
#   Target power = 80%
#   Condition-level SD = 1
#   Compound-symmetric within-participant correlation r = 0.50
#
# The full 18-condition covariance structure is used to derive the
# marginal covariance structure for each primary main effect.
#
# Power is calculated analytically. No simulated data or random seed
# are required. Results are independently verified using the
# corresponding noncentral-F formulation.
# ======================================================================


# ----------------------------------------------------------------------
# 1. Package and analysis specifications
# ----------------------------------------------------------------------

library(Superpower)

cohen_f <- 0.25
```

```
alpha_level <- 0.05
target_power <- 80
sd_condition <- 1
r_condition <- 0.50

cat("===================================================================\n")
cat("SOFTWARE\n")
cat("===================================================================\n")
cat("R version:", R.version.string, "\n")
cat(
  "Superpower version:",
  as.character(packageVersion("Superpower")),
  "\n\n"
)


# ---------------------------------------------------------------------
# 2. Full 3 x 3 x 2 repeated-measures design
# ---------------------------------------------------------------------

condition_grid <- expand.grid(
  gear_ratio = 1:2,
  cadence = 1:3,
  posture = 1:3
)

condition_names <- with(
  condition_grid,
  paste0(
    "P", posture,
    "_C", cadence,
```

```
    "_G", gear_ratio
  )
)

n_conditions <- nrow(condition_grid)

stopifnot(n_conditions == 18)



# ----------------------------------------------------------------------
# 3. Full 18 x 18 covariance matrix
# ----------------------------------------------------------------------
#
# The condition-level variance is standardized to 1.
# All pairs of repeated conditions have correlation r = 0.50.
# ----------------------------------------------------------------------

Sigma18 <- matrix(
  r_condition * sd_condition^2,
  nrow = n_conditions,
  ncol = n_conditions
)

diag(Sigma18) <- sd_condition^2

dimnames(Sigma18) <- list(
  condition_names,
  condition_names
)
```

```
# ----------------------------------------------------------------------
# 4. Averaging matrices for the primary main effects
# ----------------------------------------------------------------------
#
# Posture:
#   each level averages 3 cadence x 2 gear-ratio conditions = 6
#
# Cadence:
#   each level averages 3 posture x 2 gear-ratio conditions = 6
#
# Gear ratio:
#   each level averages 3 posture x 3 cadence conditions = 9
# ----------------------------------------------------------------------

make_average_matrix <- function(factor_values) {

  factor_levels <- sort(unique(factor_values))

  A <- matrix(
    0,
    nrow = length(factor_levels),
    ncol = length(factor_values)
  )

  for (i in seq_along(factor_levels)) {

    idx <- factor_values == factor_levels[i]

    A[i, idx] <- 1 / sum(idx)
  }
```

```
  A
}



A_posture <- make_average_matrix(
  condition_grid$posture
)

A_cadence <- make_average_matrix(
  condition_grid$cadence
)

A_gear_ratio <- make_average_matrix(
  condition_grid$gear_ratio
)

A_list <- list(
  posture = A_posture,
  cadence = A_cadence,
  gear_ratio = A_gear_ratio
)



# ----------------------------------------------------------------------
# 5. Main-effect mean patterns corresponding to Cohen's f = 0.25
# ----------------------------------------------------------------------
#
# Cohen's f is defined relative to the standardized condition-level SD.
#
# For a three-level symmetric effect:
#   means = (-a, 0, +a)
```

```
#   a = f * sqrt(3/2)
#
# For a two-level effect:
#   means = (-f, +f)
# --------------------------------------------------------------------

a_three <- cohen_f * sqrt(3 / 2)

mu_list <- list(

  posture = c(
    -a_three,
    0,
    a_three
  ),

  cadence = c(
    -a_three,
    0,
    a_three
  ),

  gear_ratio = c(
    -cohen_f,
    cohen_f
  )
)


calculate_cohen_f <- function(mu, sd = 1) {
```

```
  sqrt(
    mean(
      (mu - mean(mu))^2
    )
  ) / sd
}


f_check <- vapply(
  mu_list,
  calculate_cohen_f,
  numeric(1),
  sd = sd_condition
)

cat("============================================================\n")
cat("EFFECT-SIZE VERIFICATION\n")
cat("============================================================\n")

print(f_check)
cat("\n")

stopifnot(
  all(
    abs(f_check - cohen_f) < 1e-12
  )
)


# ----------------------------------------------------------------------
# 6. Derive marginal covariance structures
```

```
# ----------------------------------------------------------------------

derive_marginal_parameters <- function(effect) {

  A <- A_list[[effect]]

  Sigma_marginal <-
    A %*%
    Sigma18 %*%
    t(A)

  Sigma_marginal <-
    (
      Sigma_marginal +
        t(Sigma_marginal)
    ) / 2

  marginal_sd <- sqrt(
    diag(Sigma_marginal)
  )

  marginal_cor <- cov2cor(
    Sigma_marginal
  )

  off_diagonal_cor <-
    marginal_cor[
      upper.tri(marginal_cor)
    ]

  stopifnot(
```

```
    max(marginal_sd) -
      min(marginal_sd) <
      1e-12
  )

  stopifnot(
    max(off_diagonal_cor) -
      min(off_diagonal_cor) <
      1e-12
  )

  list(
    covariance = Sigma_marginal,
    sd = marginal_sd[1],
    r = off_diagonal_cor[1],
    conditions_averaged =
      sum(A[1, ] > 0)
  )
}


marginal_parameters <- lapply(
  names(A_list),
  derive_marginal_parameters
)

names(marginal_parameters) <- names(A_list)


marginal_summary <- data.frame(
```

```
  effect = c(
    "Posture",
    "Cadence",
    "Gear ratio"
  ),

  conditions_averaged = c(
    marginal_parameters$posture$conditions_averaged,
    marginal_parameters$cadence$conditions_averaged,
    marginal_parameters$gear_ratio$conditions_averaged
  ),

  marginal_variance = c(
    marginal_parameters$posture$sd^2,
    marginal_parameters$cadence$sd^2,
    marginal_parameters$gear_ratio$sd^2
  ),

  marginal_SD = c(
    marginal_parameters$posture$sd,
    marginal_parameters$cadence$sd,
    marginal_parameters$gear_ratio$sd
  ),

  marginal_correlation = c(
    marginal_parameters$posture$r,
    marginal_parameters$cadence$r,
    marginal_parameters$gear_ratio$r
  )
)
```

```r
cat("============================================================\n")
cat("MARGINAL DESIGN PARAMETERS\n")
cat("============================================================\n")

print(
  marginal_summary,
  row.names = FALSE,
  digits = 8
)

cat("\n")


# ----------------------------------------------------------------------
# 7. Primary deterministic analytical power calculation
# ----------------------------------------------------------------------

get_power <- function(effect, n) {

  mu <- mu_list[[effect]]

  marginal <-
    marginal_parameters[[effect]]

  n_levels <- length(mu)

  design_result <- ANOVA_design(
    design = paste0(
      n_levels,
      "w"
```

```
   ),
   n = n,
   mu = mu,
   sd = marginal$sd,
   r = marginal$r,
   plot = FALSE
  )

  power_result <- power_oneway_within(
   design_result,
   alpha_level = alpha_level
  )

  as.numeric(
   power_result$power
  )
}


# ----------------------------------------------------------------------
# 8. Calculate power across candidate sample sizes
# ----------------------------------------------------------------------

effects <- c(
  "posture",
  "cadence",
  "gear_ratio"
)

candidate_n <- 3:50
```

```
power_table <- do.call(
  rbind,
  lapply(
    effects,
    function(effect_name) {

      data.frame(
        effect = effect_name,
        n = candidate_n,
        power = vapply(
          candidate_n,
          function(n) {
            get_power(
              effect = effect_name,
              n = n
            )
          },
          numeric(1)
        )
      )
    }
  )
)


# ----------------------------------------------------------------------
# 9. Minimum sample size reaching 80% power
# ----------------------------------------------------------------------

minimum_results <- do.call(
  rbind,
```

```
  lapply(
    effects,
    function(effect_name) {

      x <- power_table[
        power_table$effect ==
          effect_name,
      ]

      idx <- which(
        x$power >= target_power
      )[1]

      data.frame(
        effect = effect_name,
        required_n = x$n[idx],
        power_previous_n =
          if (idx > 1) {
            x$power[idx - 1]
          } else {
            NA_real_
          },
        power_required_n =
          x$power[idx]
      )
    }
  )
)

rownames(minimum_results) <- NULL
```

```
cat("============================================================\n")
cat("MINIMUM SAMPLE SIZE FOR 80% POWER\n")
cat("============================================================\n")

print(
  minimum_results,
  row.names = FALSE,
  digits = 8
)

cat("\n")


# ----------------------------------------------------------------------
# 10. Power around the threshold
# ----------------------------------------------------------------------

threshold_table <- power_table[
  power_table$n >= 3 &
    power_table$n <= 12,
]

cat("============================================================\n")
cat("POWER FOR n = 3 TO 12\n")
cat("============================================================\n")

print(
  threshold_table,
  row.names = FALSE,
  digits = 8
```

```
)

cat("\n")


# ---------------------------------------------------------------------
# 11. Independent noncentral-F verification
# ---------------------------------------------------------------------
#
# This independently implements the analytical repeated-measures
# noncentral-F calculation and compares it against the Superpower
# calculation above.
# ---------------------------------------------------------------------

power_ncf <- function(effect, n) {

  mu <- mu_list[[effect]]

  marginal <-
    marginal_parameters[[effect]]

  k <- length(mu)

  f_marginal <- sqrt(
    mean(
      (mu - mean(mu))^2
    )
  ) / marginal$sd

  df1 <- k - 1
```

```
  df2 <- (
    n - 1
  ) * (
    k - 1
  )

  lambda <- (
    f_marginal^2 *
      n *
      k
  ) / (
    1 - marginal$r
  )

  Fcrit <- qf(
    1 - alpha_level,
    df1 = df1,
    df2 = df2
  )

  100 * pf(
    Fcrit,
    df1 = df1,
    df2 = df2,
    ncp = lambda,
    lower.tail = FALSE
  )
}


verification_table <- power_table
```

```
verification_table$power_ncf <- mapply(
  power_ncf,
  effect = verification_table$effect,
  n = verification_table$n
)

verification_table$difference <-
  verification_table$power -
  verification_table$power_ncf

max_absolute_difference <- max(
  abs(
    verification_table$difference
  )
)


cat("============================================================\n")
cat("INDEPENDENT ANALYTICAL VERIFICATION\n")
cat("============================================================\n")

cat(
  "Maximum absolute difference between Superpower and",
  "independent noncentral-F calculation:",
  format(
    max_absolute_difference,
    scientific = TRUE
  ),
  "\n"
)
```

```
stopifnot(
  max_absolute_difference < 1e-8
)

cat("VERIFICATION PASSED\n\n")



# ----------------------------------------------------------------------
# 12. Final analysis specifications
# ----------------------------------------------------------------------

cat("============================================================\n")
cat("ANALYSIS SPECIFICATIONS\n")
cat("============================================================\n")

cat(
  "Full design: 3 posture x 3 cadence x 2 gear ratio; all repeated\n"
)

cat(
  "Number of repeated conditions:",
  n_conditions,
  "\n"
)

cat(
  "Cohen's f:",
  cohen_f,
  "\n"
)
```

```
cat(
  "Condition-level SD:",
  sd_condition,
  "\n"
)

cat(
  "Compound-symmetric correlation:",
  r_condition,
  "\n"
)

cat(
  "Nominal alpha:",
  alpha_level,
  "\n"
)

cat(
  "Target power:",
  target_power,
  "%\n"
)

cat(
  "Simulation used: NO\n"
)

cat(
  "Random seed required: NO\n"
```

```
)

cat(
  "Superpower version:",
  as.character(
    packageVersion("Superpower")
  ),
  "\n"
)
```
