# Supplementary material for: Multi-Location Analysis of Cycling Forces and Impulses Across Posture, Cadence, and Gear Ratio
Source: Sensors (Basel). 2026 Oct 16;26(20):6559. doi: 10.3390/s26206559 (PMC13708784; doi:10.3390/s26206559)
Supplement: Supplementary file 1 [file sensors-26-06559-s001.zip › Tables S1-S4_Descriptive results.pdf]

Supplementary Descriptive Results

Tables S1–S4 provide sex-stratified descriptive statistics (mean ± SD) for body-mass-normalized peak vertical force (N/kg), cycle-averaged vertical force (N/kg), and vertical impulse per cycle (N·s/kg).

Note: The same outcome-specific data exclusions as in the inferential analyses were applied.

Table S1. Male descriptive statistics for body-mass-normalized force outcomes on the original measurement scale.

Panel A. Peak vertical force (N/kg).

Posture	Cadence	Gear ratio	Left grip	Right grip	Left pedal	Right pedal	Saddle
comfort	slow	light	1.032 ± 0.153	1.056 ± 0.156	2.541 ± 0.238	2.548 ± 0.296	3.910 ± 0.980
comfort	slow	heavy	0.970 ± 0.164	0.974 ± 0.164	3.910 ± 0.378	3.953 ± 0.172	3.120 ± 0.846
comfort	self-selected	light	1.028 ± 0.165	1.022 ± 0.180	2.655 ± 0.274	2.659 ± 0.258	3.888 ± 1.041
comfort	self-selected	heavy	0.968 ± 0.172	0.952 ± 0.181	4.076 ± 0.486	4.010 ± 0.345	3.156 ± 0.962
comfort	quick	light	1.049 ± 0.133	1.027 ± 0.160	2.869 ± 0.447	2.695 ± 0.407	3.924 ± 0.866
comfort	quick	heavy	0.966 ± 0.173	0.956 ± 0.182	4.329 ± 0.287	4.255 ± 0.285	3.003 ± 1.011
neutral	slow	light	1.099 ± 0.173	1.093 ± 0.169	2.548 ± 0.280	2.589 ± 0.275	3.957 ± 0.598
neutral	slow	heavy	1.050 ± 0.170	1.027 ± 0.190	3.891 ± 0.290	3.929 ± 0.163	3.185 ± 1.057
neutral	self-selected	light	1.097 ± 0.152	1.085 ± 0.176	2.701 ± 0.323	2.744 ± 0.266	4.094 ± 0.602
neutral	self-selected	heavy	1.029 ± 0.161	0.997 ± 0.181	3.958 ± 0.330	4.075 ± 0.322	3.150 ± 1.018
neutral	quick	light	1.133 ± 0.190	1.050 ± 0.194	2.883 ± 0.454	2.822 ± 0.331	4.013 ± 0.653
neutral	quick	heavy	1.010 ± 0.156	1.004 ± 0.167	4.243 ± 0.426	4.290 ± 0.342	3.241 ± 0.872
athletic	slow	light	1.109 ± 0.155	1.128 ± 0.166	2.556 ± 0.345	2.449 ± 0.228	3.810 ± 0.467
athletic	slow	heavy	1.016 ± 0.120	1.033 ± 0.162	3.889 ± 0.311	3.943 ± 0.333	3.059 ± 0.852
athletic	self-selected	light	1.109 ± 0.148	1.150 ± 0.163	2.790 ± 0.456	2.809 ± 0.296	3.654 ± 0.710
athletic	self-selected	heavy	0.997 ± 0.166	1.029 ± 0.150	4.153 ± 0.377	4.219 ± 0.265	2.971 ± 0.821
athletic	quick	light	1.144 ± 0.177	1.123 ± 0.154	2.984 ± 0.508	2.889 ± 0.289	3.945 ± 0.669
athletic	quick	heavy	1.020 ± 0.176	1.031 ± 0.187	4.220 ± 0.472	4.366 ± 0.243	2.965 ± 0.983

Panel B. Cycle-averaged vertical force (N/kg).

Posture	Cadence	Gear ratio	Left grip	Right grip	Left pedal	Right pedal	Saddle
comfort	slow	light	0.486 ± 0.080	0.486 ± 0.057	0.835 ± 0.077	0.859 ± 0.057	1.783 ± 0.524
comfort	slow	heavy	0.452 ± 0.085	0.423 ± 0.076	1.398 ± 0.134	1.396 ± 0.057	1.408 ± 0.474
comfort	self-selected	light	0.493 ± 0.078	0.479 ± 0.072	0.834 ± 0.060	0.878 ± 0.039	1.780 ± 0.564
comfort	self-selected	heavy	0.466 ± 0.087	0.412 ± 0.073	1.447 ± 0.165	1.406 ± 0.139	1.430 ± 0.501
comfort	quick	light	0.496 ± 0.062	0.471 ± 0.052	0.878 ± 0.084	0.851 ± 0.110	1.764 ± 0.465
comfort	quick	heavy	0.451 ± 0.074	0.414 ± 0.064	1.492 ± 0.124	1.471 ± 0.129	1.352 ± 0.523
neutral	slow	light	0.523 ± 0.079	0.504 ± 0.072	0.853 ± 0.082	0.866 ± 0.045	1.874 ± 0.379
neutral	slow	heavy	0.487 ± 0.075	0.443 ± 0.085	1.396 ± 0.117	1.394 ± 0.074	1.411 ± 0.566
neutral	self-selected	light	0.535 ± 0.072	0.510 ± 0.096	0.863 ± 0.065	0.890 ± 0.050	1.904 ± 0.343
neutral	self-selected	heavy	0.483 ± 0.065	0.430 ± 0.080	1.419 ± 0.122	1.411 ± 0.149	1.437 ± 0.508
neutral	quick	light	0.548 ± 0.088	0.497 ± 0.080	0.880 ± 0.068	0.885 ± 0.053	1.856 ± 0.363
neutral	quick	heavy	0.481 ± 0.070	0.428 ± 0.082	1.492 ± 0.163	1.484 ± 0.163	1.458 ± 0.453
athletic	slow	light	0.512 ± 0.073	0.499 ± 0.063	0.838 ± 0.082	0.765 ± 0.169	1.761 ± 0.252
athletic	slow	heavy	0.461 ± 0.072	0.440 ± 0.080	1.396 ± 0.109	1.347 ± 0.150	1.341 ± 0.474
athletic	self-selected	light	0.533 ± 0.076	0.531 ± 0.068	0.886 ± 0.093	0.908 ± 0.069	1.718 ± 0.321
athletic	self-selected	heavy	0.454 ± 0.073	0.434 ± 0.056	1.463 ± 0.133	1.448 ± 0.125	1.268 ± 0.413
athletic	quick	light	0.550 ± 0.066	0.517 ± 0.055	0.914 ± 0.100	0.922 ± 0.079	1.802 ± 0.333
athletic	quick	heavy	0.458 ± 0.086	0.435 ± 0.094	1.502 ± 0.216	1.494 ± 0.179	1.273 ± 0.468

Note: For males, handlebar and saddle outcomes used n = 10; pedal force outcomes used n = 6 after exclusion of four duplicated pedal recordings. Cycle-averaged pedal force inherited the exclusions applied to the impulse data from which it was derived.

Table S2. Female descriptive statistics for body-mass-normalized force outcomes on the original measurement scale.

Panel A. Peak vertical force (N/kg).

Posture	Cadence	Gear ratio	Left grip	Right grip	Left pedal	Right pedal	Saddle
comfort	slow	light	1.145 ± 0.305	0.966 ± 0.155	2.728 ± 0.351	2.647 ± 0.399	5.603 ± 1.396
comfort	slow	heavy	1.152 ± 0.318	0.924 ± 0.144	4.386 ± 0.718	4.193 ± 0.729	4.890 ± 1.183
comfort	self-selected	light	1.209 ± 0.333	0.979 ± 0.159	2.939 ± 0.288	2.966 ± 0.534	5.772 ± 1.339
comfort	self-selected	heavy	1.143 ± 0.338	0.904 ± 0.127	4.618 ± 0.742	4.586 ± 0.729	4.634 ± 1.079
comfort	quick	light	1.139 ± 0.332	0.963 ± 0.151	3.183 ± 0.467	3.082 ± 0.461	5.649 ± 1.351
comfort	quick	heavy	1.128 ± 0.348	0.919 ± 0.167	4.851 ± 0.574	4.735 ± 0.785	4.716 ± 1.069
neutral	slow	light	1.249 ± 0.350	1.016 ± 0.123	2.666 ± 0.282	2.706 ± 0.513	5.382 ± 1.342
neutral	slow	heavy	1.222 ± 0.333	1.000 ± 0.132	4.302 ± 0.634	4.221 ± 0.771	4.733 ± 1.261
neutral	self-selected	light	1.254 ± 0.336	1.037 ± 0.154	2.945 ± 0.326	2.993 ± 0.521	5.526 ± 1.368
neutral	self-selected	heavy	1.210 ± 0.350	0.999 ± 0.171	4.462 ± 0.422	4.728 ± 0.777	4.683 ± 1.396
neutral	quick	light	1.213 ± 0.366	1.063 ± 0.126	3.145 ± 0.674	3.003 ± 0.516	5.636 ± 1.511
neutral	quick	heavy	1.187 ± 0.285	0.966 ± 0.121	4.894 ± 0.556	4.790 ± 0.523	4.601 ± 0.912
athletic	slow	light	1.249 ± 0.359	1.012 ± 0.203	2.672 ± 0.318	2.666 ± 0.414	5.429 ± 1.216
athletic	slow	heavy	1.196 ± 0.322	0.993 ± 0.151	4.197 ± 0.601	4.158 ± 0.747	4.769 ± 0.938
athletic	self-selected	light	1.238 ± 0.374	1.043 ± 0.188	2.875 ± 0.376	2.802 ± 0.441	5.338 ± 1.086
athletic	self-selected	heavy	1.195 ± 0.270	0.956 ± 0.153	4.560 ± 0.434	4.532 ± 0.722	4.671 ± 1.065
athletic	quick	light	1.250 ± 0.329	1.052 ± 0.168	3.153 ± 0.544	2.921 ± 0.439	5.487 ± 1.183
athletic	quick	heavy	1.180 ± 0.298	0.939 ± 0.161	4.915 ± 0.647	4.786 ± 0.695	4.734 ± 0.973

Panel B. Cycle-averaged vertical force (N/kg).

Posture	Cadence	Gear ratio	Left grip	Right grip	Left pedal	Right pedal	Saddle
comfort	slow	light	0.517 ± 0.176	0.431 ± 0.083	0.925 ± 0.104	0.925 ± 0.073	2.593 ± 0.669
comfort	slow	heavy	0.475 ± 0.187	0.396 ± 0.062	1.582 ± 0.190	1.532 ± 0.132	2.122 ± 0.586
comfort	self-selected	light	0.559 ± 0.204	0.459 ± 0.082	0.982 ± 0.093	1.004 ± 0.096	2.737 ± 0.696
comfort	self-selected	heavy	0.480 ± 0.169	0.387 ± 0.063	1.636 ± 0.201	1.577 ± 0.196	1.986 ± 0.520
comfort	quick	light	0.540 ± 0.217	0.454 ± 0.091	0.993 ± 0.150	1.021 ± 0.108	2.677 ± 0.681
comfort	quick	heavy	0.468 ± 0.203	0.364 ± 0.063	1.664 ± 0.152	1.666 ± 0.153	2.073 ± 0.474
neutral	slow	light	0.597 ± 0.194	0.458 ± 0.064	0.939 ± 0.083	0.982 ± 0.108	2.570 ± 0.738
neutral	slow	heavy	0.526 ± 0.186	0.422 ± 0.055	1.548 ± 0.176	1.577 ± 0.191	2.090 ± 0.584
neutral	self-selected	light	0.580 ± 0.205	0.488 ± 0.083	0.964 ± 0.079	1.020 ± 0.100	2.607 ± 0.662
neutral	self-selected	heavy	0.511 ± 0.174	0.417 ± 0.066	1.617 ± 0.168	1.704 ± 0.208	2.071 ± 0.625
neutral	quick	light	0.567 ± 0.217	0.496 ± 0.083	0.994 ± 0.167	1.011 ± 0.107	2.675 ± 0.780
neutral	quick	heavy	0.507 ± 0.186	0.403 ± 0.073	1.698 ± 0.143	1.699 ± 0.098	1.999 ± 0.446
athletic	slow	light	0.562 ± 0.195	0.457 ± 0.103	0.939 ± 0.087	0.962 ± 0.107	2.516 ± 0.613
athletic	slow	heavy	0.524 ± 0.179	0.437 ± 0.070	1.572 ± 0.176	1.551 ± 0.157	2.075 ± 0.429
athletic	self-selected	light	0.570 ± 0.193	0.482 ± 0.088	0.962 ± 0.093	0.955 ± 0.113	2.506 ± 0.563
athletic	self-selected	heavy	0.504 ± 0.190	0.400 ± 0.060	1.645 ± 0.104	1.626 ± 0.127	2.039 ± 0.475
athletic	quick	light	0.592 ± 0.208	0.497 ± 0.081	1.015 ± 0.146	0.993 ± 0.072	2.571 ± 0.566
athletic	quick	heavy	0.504 ± 0.181	0.381 ± 0.068	1.710 ± 0.160	1.694 ± 0.112	2.043 ± 0.458

Note: For females, peak-force outcomes used n = 10 at all locations. Cycle-averaged force used n = 10 for handlebar and saddle outcomes and n = 9 for pedal outcomes because one participant had missing left-pedal impulse data and one erroneous right-pedal impulse value, leading to outcome-specific exclusion.

Table S3. Male descriptive statistics for body-mass-normalized impulse-per-cycle outcomes on the original measurement scale.

Posture	Cadence	Gear ratio	Left grip	Right grip	Left pedal	Right pedal	Saddle
comfort	slow	light	0.409 ± 0.082	0.408 ± 0.062	0.688 ± 0.102	0.705 ± 0.061	1.498 ± 0.461
comfort	slow	heavy	0.381 ± 0.086	0.354 ± 0.066	1.154 ± 0.175	1.148 ± 0.112	1.179 ± 0.397
comfort	self-selected	light	0.364 ± 0.071	0.353 ± 0.063	0.604 ± 0.070	0.634 ± 0.043	1.309 ± 0.424
comfort	self-selected	heavy	0.344 ± 0.072	0.301 ± 0.050	1.049 ± 0.164	1.019 ± 0.147	1.046 ± 0.348
comfort	quick	light	0.326 ± 0.054	0.308 ± 0.039	0.567 ± 0.075	0.550 ± 0.090	1.155 ± 0.311
comfort	quick	heavy	0.295 ± 0.051	0.271 ± 0.044	0.965 ± 0.127	0.952 ± 0.129	0.884 ± 0.338
neutral	slow	light	0.441 ± 0.088	0.422 ± 0.068	0.703 ± 0.104	0.712 ± 0.064	1.572 ± 0.351
neutral	slow	heavy	0.409 ± 0.074	0.370 ± 0.070	1.150 ± 0.152	1.147 ± 0.119	1.187 ± 0.485
neutral	self-selected	light	0.395 ± 0.071	0.375 ± 0.074	0.625 ± 0.073	0.643 ± 0.061	1.398 ± 0.268
neutral	self-selected	heavy	0.356 ± 0.063	0.316 ± 0.061	1.027 ± 0.131	1.022 ± 0.146	1.057 ± 0.377
neutral	quick	light	0.360 ± 0.070	0.326 ± 0.057	0.569 ± 0.068	0.571 ± 0.057	1.214 ± 0.250
neutral	quick	heavy	0.316 ± 0.058	0.279 ± 0.051	0.965 ± 0.147	0.960 ± 0.144	0.952 ± 0.291
athletic	slow	light	0.431 ± 0.081	0.418 ± 0.058	0.691 ± 0.098	0.636 ± 0.174	1.472 ± 0.208
athletic	slow	heavy	0.389 ± 0.079	0.367 ± 0.061	1.150 ± 0.144	1.112 ± 0.183	1.123 ± 0.392
athletic	self-selected	light	0.393 ± 0.069	0.390 ± 0.055	0.642 ± 0.094	0.657 ± 0.079	1.259 ± 0.222
athletic	self-selected	heavy	0.334 ± 0.061	0.319 ± 0.044	1.061 ± 0.152	1.050 ± 0.146	0.930 ± 0.297
athletic	quick	light	0.361 ± 0.056	0.338 ± 0.038	0.591 ± 0.089	0.595 ± 0.074	1.179 ± 0.223
athletic	quick	heavy	0.302 ± 0.066	0.284 ± 0.062	0.974 ± 0.182	0.968 ± 0.162	0.830 ± 0.294

Note: For males, handlebar and saddle outcomes used n = 10; pedal impulse outcomes used n = 6 after exclusion of four duplicated pedal recordings.

Table S4. Female descriptive statistics for body-mass-normalized impulse-per-cycle outcomes on the original measurement scale.

Posture	Cadence	Gear ratio	Left grip	Right grip	Left pedal	Right pedal	Saddle
comfort	slow	light	0.545 ± 0.251	0.445 ± 0.098	0.961 ± 0.166	0.964 ± 0.167	2.712 ± 0.972
comfort	slow	heavy	0.503 ± 0.275	0.411 ± 0.089	1.647 ± 0.324	1.596 ± 0.291	2.231 ± 0.847
comfort	self-selected	light	0.499 ± 0.229	0.403 ± 0.080	0.873 ± 0.174	0.886 ± 0.120	2.424 ± 0.749
comfort	self-selected	heavy	0.428 ± 0.186	0.341 ± 0.067	1.442 ± 0.216	1.383 ± 0.141	1.780 ± 0.641
comfort	quick	light	0.421 ± 0.206	0.347 ± 0.066	0.760 ± 0.112	0.787 ± 0.131	2.063 ± 0.622
comfort	quick	heavy	0.365 ± 0.192	0.278 ± 0.047	1.277 ± 0.156	1.275 ± 0.117	1.610 ± 0.519
neutral	slow	light	0.632 ± 0.291	0.475 ± 0.095	0.984 ± 0.225	1.025 ± 0.200	2.698 ± 1.037
neutral	slow	heavy	0.558 ± 0.276	0.438 ± 0.086	1.608 ± 0.289	1.636 ± 0.281	2.198 ± 0.820
neutral	self-selected	light	0.519 ± 0.235	0.428 ± 0.072	0.856 ± 0.160	0.903 ± 0.149	2.326 ± 0.812
neutral	self-selected	heavy	0.458 ± 0.202	0.369 ± 0.086	1.429 ± 0.231	1.506 ± 0.247	1.861 ± 0.742
neutral	quick	light	0.442 ± 0.206	0.381 ± 0.078	0.761 ± 0.128	0.775 ± 0.090	2.078 ± 0.782
neutral	quick	heavy	0.395 ± 0.181	0.310 ± 0.070	1.306 ± 0.181	1.311 ± 0.203	1.556 ± 0.506
athletic	slow	light	0.596 ± 0.296	0.472 ± 0.119	0.977 ± 0.168	1.006 ± 0.225	2.617 ± 0.812
athletic	slow	heavy	0.556 ± 0.272	0.456 ± 0.112	1.629 ± 0.252	1.615 ± 0.302	2.169 ± 0.629
athletic	self-selected	light	0.513 ± 0.240	0.424 ± 0.084	0.853 ± 0.164	0.846 ± 0.155	2.215 ± 0.608
athletic	self-selected	heavy	0.453 ± 0.219	0.356 ± 0.090	1.457 ± 0.224	1.436 ± 0.196	1.818 ± 0.573
athletic	quick	light	0.461 ± 0.209	0.382 ± 0.076	0.779 ± 0.127	0.766 ± 0.120	1.983 ± 0.536
athletic	quick	heavy	0.392 ± 0.170	0.295 ± 0.072	1.317 ± 0.198	1.306 ± 0.192	1.576 ± 0.435

Note: For females, handlebar and saddle outcomes used n = 10; left- and right-pedal impulse outcomes used n = 9 because one participant had missing left-pedal impulse data and one erroneous right-pedal impulse value, both leading to outcome-specific exclusion.
