# Supplementary material for: Multi-Location Analysis of Cycling Forces and Impulses Across Posture, Cadence, and Gear Ratio
Source: Sensors (Basel). 2026 Oct 16;26(20):6559. doi: 10.3390/s26206559 (PMC13708784; doi:10.3390/s26206559)
Supplement: Supplementary file 1 [file sensors-26-06559-s001.zip › Tables S5-S13_Inferential results.pdf]

Supplementary Inferential Results

Tables S5–S13 provide the complete inferential statistics for the final body-mass-normalized 3 × 3 × 2 repeated-measures analyses, follow-up comparisons, simple-effects analyses, and natural-log-transformed sensitivity analyses.

Note: Vertical peak force and cycle-averaged vertical force are reported in N/kg. Vertical impulse per cycle is reported in N·s/kg. For omnibus tests, p denotes the unadjusted p value after any required sphericity correction, and pHolm denotes the Holm-adjusted p value across all 15 outcomes separately for each factorial effect. For post hoc and simple-effects comparisons, pHolm denotes the Holm-adjusted p value within the relevant follow-up family. GG = Greenhouse-Geisser; HF = Huynh-Feldt; ηp^2 = partial eta squared. Values in brackets are 95% confidence intervals. Confidence intervals for ηp^2 were obtained by inversion of the noncentral-F distribution. For paired comparisons, the reported F statistic is equivalent to t^2 with df1 = 1. All p values are reported numerically without threshold notation.

Table S5. Complete primary 3 × 3 × 2 repeated-measures ANOVA results on the original measurement scale.

Outcome	n	Effect	Corr.	F	df1	df2	p	pHolm	ηp² [95% CI]
Left grip peak force	20	Posture	None	19.883	2.000	38.000	1.232558e-06	1.848837e-05	0.511 [0.270, 0.662]
Left grip peak force	20	Cadence	GG	0.244	1.462	27.779	0.715434	1.000000	0.013 [0.000, 0.163]
Left grip peak force	20	Gear ratio	None	27.839	1.000	19.000	4.305531e-05	8.611062e-05	0.594 [0.272, 0.762]
Left grip peak force	20	Posture × cadence	None	2.066	4.000	76.000	0.093506	1.000000	0.098 [0.000, 0.208]
Left grip peak force	20	Posture × gear ratio	None	3.860	2.000	38.000	0.029770	0.446552	0.169 [0.000, 0.366]
Left grip peak force	20	Cadence × gear ratio	None	2.313	2.000	38.000	0.112786	0.789501	0.109 [0.000, 0.295]
Left grip peak force	20	Posture × cadence × gear ratio	None	0.453	4.000	76.000	0.770105	1.000000	0.023 [0.000, 0.074]
Left grip impulse per cycle	20	Posture	None	15.855	2.000	38.000	9.848486e-06	0.000128	0.455 [0.207, 0.619]
Left grip impulse per cycle	20	Cadence	GG	48.393	1.095	20.810	4.588104e-07	4.588104e-06	0.718 [0.469, 0.833]
Left grip impulse per cycle	20	Gear ratio	None	57.492	1.000	19.000	3.681664e-07	1.104499e-06	0.752 [0.510, 0.856]
Left grip impulse per cycle	20	Posture × cadence	None	3.538	4.000	76.000	0.010587	0.158804	0.157 [0.011, 0.283]
Left grip impulse per cycle	20	Posture × gear ratio	GG	0.930	1.311	24.909	0.370126	1.000000	0.047 [0.000, 0.264]
Left grip impulse per cycle	20	Cadence × gear ratio	None	0.771	2.000	38.000	0.469854	1.000000	0.039 [0.000, 0.186]
Left grip impulse per cycle	20	Posture × cadence × gear ratio	None	0.818	4.000	76.000	0.517791	1.000000	0.041 [0.000, 0.115]
Right grip peak force	20	Posture	GG	23.443	1.449	27.528	6.964407e-06	9.750170e-05	0.552 [0.277, 0.712]
Right grip peak force	20	Cadence	None	1.214	2.000	38.000	0.308254	1.000000	0.060 [0.000, 0.225]
Right grip peak force	20	Gear ratio	None	27.689	1.000	19.000	4.442977e-05	8.611062e-05	0.593 [0.270, 0.762]
Right grip peak force	20	Posture × cadence	GG	0.385	2.549	48.436	0.732213	1.000000	0.020 [0.000, 0.108]
Right grip peak force	20	Posture × gear ratio	HF	1.862	1.636	31.091	0.177583	1.000000	0.089 [0.000, 0.296]
Right grip peak force	20	Cadence × gear ratio	None	2.061	2.000	38.000	0.141374	0.789501	0.098 [0.000, 0.281]

Outcome	n	Effect	Corr.	F	df1	df2	p	pHolm	ηp² [95% CI]
Right grip peak force	20	Posture × cadence × gear ratio	None	0.656	4.000	76.000	0.624394	1.000000	0.033 [0.000, 0.099]
Right grip impulse per cycle	20	Posture	None	10.699	2.000	38.000	0.000206	0.002062	0.360 [0.115, 0.544]
Right grip impulse per cycle	20	Cadence	GG	188.300	1.222	23.218	2.412267e-13	3.618401e-12	0.908 [0.822, 0.945]
Right grip impulse per cycle	20	Gear ratio	None	67.967	1.000	19.000	1.069157e-07	5.270921e-07	0.782 [0.563, 0.874]
Right grip impulse per cycle	20	Posture × cadence	None	0.013	4.000	76.000	0.999639	1.000000	0.001 [0.000, 0.000]
Right grip impulse per cycle	20	Posture × gear ratio	HF	0.395	1.672	31.770	0.640380	1.000000	0.020 [0.000, 0.167]
Right grip impulse per cycle	20	Cadence × gear ratio	None	4.665	2.000	38.000	0.015430	0.185166	0.197 [0.008, 0.396]
Right grip impulse per cycle	20	Posture × cadence × gear ratio	None	0.902	4.000	76.000	0.466944	1.000000	0.045 [0.000, 0.123]
Left pedal peak force	16	Posture	None	0.460	2.000	30.000	0.635504	1.000000	0.030 [0.000, 0.183]
Left pedal peak force	16	Cadence	GG	60.473	1.384	20.759	2.034127e-08	2.644366e-07	0.801 [0.609, 0.882]
Left pedal peak force	16	Gear ratio	None	308.688	1.000	15.000	2.043954e-11	2.452745e-10	0.954 [0.890, 0.975]
Left pedal peak force	16	Posture × cadence	GG	0.757	1.924	28.867	0.473263	1.000000	0.048 [0.000, 0.229]
Left pedal peak force	16	Posture × gear ratio	None	0.701	2.000	30.000	0.503839	1.000000	0.045 [0.000, 0.217]
Left pedal peak force	16	Cadence × gear ratio	None	0.652	2.000	30.000	0.528294	1.000000	0.042 [0.000, 0.210]
Left pedal peak force	16	Posture × cadence × gear ratio	None	1.577	4.000	60.000	0.192174	1.000000	0.095 [0.000, 0.214]
Left pedal impulse per cycle	15	Posture	None	1.248	2.000	28.000	0.302682	1.000000	0.082 [0.000, 0.286]
Left pedal impulse per cycle	15	Cadence	GG	51.345	1.303	18.235	2.826750e-07	3.109425e-06	0.786 [0.561, 0.877]
Left pedal impulse per cycle	15	Gear ratio	None	281.488	1.000	14.000	1.146673e-10	1.261340e-09	0.953 [0.883, 0.975]
Left pedal impulse per cycle	15	Posture × cadence	None	0.662	4.000	56.000	0.621244	1.000000	0.045 [0.000, 0.131]
Left pedal impulse per cycle	15	Posture × gear ratio	None	0.667	2.000	28.000	0.521331	1.000000	0.045 [0.000, 0.224]
Left pedal impulse per cycle	15	Cadence × gear ratio	None	12.526	2.000	28.000	0.000130	0.001952	0.472 [0.178, 0.652]
Left pedal impulse per cycle	15	Posture × cadence × gear ratio	None	0.780	4.000	56.000	0.542650	1.000000	0.053 [0.000, 0.146]
Right pedal peak force	16	Posture	None	1.071	2.000	30.000	0.355406	1.000000	0.067 [0.000, 0.256]
Right pedal peak force	16	Cadence	None	46.581	2.000	30.000	6.305344e-10	8.827481e-09	0.756 [0.579, 0.843]
Right pedal peak force	16	Gear ratio	None	313.237	1.000	15.000	1.840256e-11	2.392332e-10	0.954 [0.892, 0.975]
Right pedal peak force	16	Posture × cadence	None	0.452	4.000	60.000	0.770787	1.000000	0.029 [0.000, 0.092]
Right pedal peak force	16	Posture × gear ratio	GG	0.751	1.438	21.576	0.442363	1.000000	0.048 [0.000, 0.278]
Right pedal peak force	16	Cadence × gear ratio	None	4.737	2.000	30.000	0.016297	0.185166	0.240 [0.009, 0.459]
Right pedal peak force	16	Posture × cadence × gear ratio	GG	0.183	2.105	31.579	0.843883	1.000000	0.012 [0.000, 0.113]
Right pedal impulse per cycle	15	Posture	None	2.611	2.000	28.000	0.091273	0.821459	0.157 [0.000, 0.382]
Right pedal impulse per cycle	15	Cadence	GG	52.422	1.220	17.073	5.336245e-07	4.802621e-06	0.789 [0.557, 0.881]
Right pedal impulse per cycle	15	Gear ratio	None	251.821	1.000	14.000	2.410745e-10	2.410745e-09	0.947 [0.871, 0.972]

Outcome	n	Effect	Corr.	F	df1	df2	p	pHolm	ηp² [95% CI]
Right pedal impulse per cycle	15	Posture × cadence	GG	0.887	2.486	34.811	0.440934	1.000000	0.060 [0.000, 0.218]
Right pedal impulse per cycle	15	Posture × gear ratio	None	0.721	2.000	28.000	0.495083	1.000000	0.049 [0.000, 0.231]
Right pedal impulse per cycle	15	Cadence × gear ratio	None	11.838	2.000	28.000	0.000188	0.002618	0.458 [0.163, 0.642]
Right pedal impulse per cycle	15	Posture × cadence × gear ratio	None	0.749	4.000	56.000	0.562925	1.000000	0.051 [0.000, 0.142]
Saddle peak force	20	Posture	None	0.527	2.000	38.000	0.594630	1.000000	0.027 [0.000, 0.159]
Saddle peak force	20	Cadence	None	0.188	2.000	38.000	0.829525	1.000000	0.010 [0.000, 0.100]
Saddle peak force	20	Gear ratio	None	69.761	1.000	19.000	8.784868e-08	5.270921e-07	0.786 [0.571, 0.876]
Saddle peak force	20	Posture × cadence	GG	1.709	2.439	46.332	0.186318	1.000000	0.083 [0.000, 0.235]
Saddle peak force	20	Posture × gear ratio	None	0.883	2.000	38.000	0.421800	1.000000	0.044 [0.000, 0.197]
Saddle peak force	20	Cadence × gear ratio	None	2.544	2.000	38.000	0.091866	0.734928	0.118 [0.000, 0.307]
Saddle peak force	20	Posture × cadence × gear ratio	None	0.518	4.000	76.000	0.722416	1.000000	0.027 [0.000, 0.082]
Saddle impulse per cycle	20	Posture	None	1.107	2.000	38.000	0.341045	1.000000	0.055 [0.000, 0.216]
Saddle impulse per cycle	20	Cadence	GG	51.798	1.162	22.071	1.316080e-07	1.579296e-06	0.732 [0.500, 0.839]
Saddle impulse per cycle	20	Gear ratio	None	89.523	1.000	19.000	1.273690e-08	1.018952e-07	0.825 [0.643, 0.899]
Saddle impulse per cycle	20	Posture × cadence	GG	0.599	2.179	41.397	0.567854	1.000000	0.031 [0.000, 0.156]
Saddle impulse per cycle	20	Posture × gear ratio	None	0.346	2.000	38.000	0.709815	1.000000	0.018 [0.000, 0.133]
Saddle impulse per cycle	20	Cadence × gear ratio	None	0.503	2.000	38.000	0.608743	1.000000	0.026 [0.000, 0.156]
Saddle impulse per cycle	20	Posture × cadence × gear ratio	None	0.639	4.000	76.000	0.636178	1.000000	0.033 [0.000, 0.097]
Left grip cycle-averaged vertical force	20	Posture	None	15.295	2.000	38.000	1.339370e-05	0.000161	0.446 [0.197, 0.613]
Left grip cycle-averaged vertical force	20	Cadence	None	0.394	2.000	38.000	0.677186	1.000000	0.020 [0.000, 0.141]
Left grip cycle-averaged vertical force	20	Gear ratio	None	71.963	1.000	19.000	6.940050e-08	4.858035e-07	0.791 [0.580, 0.879]
Left grip cycle-averaged vertical force	20	Posture × cadence	GG	2.340	2.935	55.770	0.084432	1.000000	0.110 [0.000, 0.253]
Left grip cycle-averaged vertical force	20	Posture × gear ratio	GG	1.182	1.356	25.762	0.304774	1.000000	0.059 [0.000, 0.279]
Left grip cycle-averaged vertical force	20	Cadence × gear ratio	None	3.634	2.000	38.000	0.035959	0.323635	0.161 [0.000, 0.357]
Left grip cycle-averaged vertical force	20	Posture × cadence × gear ratio	None	0.742	4.000	76.000	0.566232	1.000000	0.038 [0.000, 0.108]
Right grip cycle-averaged vertical force	20	Posture	None	12.011	2.000	38.000	9.068140e-05	0.000997	0.387 [0.139, 0.566]
Right grip cycle-averaged vertical force	20	Cadence	HF	1.617	1.609	30.569	0.216942	1.000000	0.078 [0.000, 0.283]
Right grip cycle-averaged vertical force	20	Gear ratio	None	68.484	1.000	19.000	1.009890e-07	5.270921e-07	0.783 [0.565, 0.874]
Right grip cycle-averaged vertical force	20	Posture × cadence	None	0.215	4.000	76.000	0.929382	1.000000	0.011 [0.000, 0.031]
Right grip cycle-averaged vertical force	20	Posture × gear ratio	None	0.931	2.000	38.000	0.402908	1.000000	0.047 [0.000, 0.201]
Right grip cycle-averaged vertical force	20	Cadence × gear ratio	None	10.852	2.000	38.000	0.000187	0.002618	0.364 [0.118, 0.547]
Right grip cycle-averaged vertical force	20	Posture × cadence × gear ratio	None	0.962	4.000	76.000	0.433645	1.000000	0.048 [0.000, 0.129]

Outcome	n	Effect	Corr.	F	df1	df2	p	pHolm	ηp² [95% CI]
Left pedal cycle-averaged vertical force	15	Posture	None	1.735	2.000	28.000	0.194850	1.000000	0.110 [0.000, 0.326]
Left pedal cycle-averaged vertical force	15	Cadence	GG	16.722	1.382	19.350	0.000220	0.001539	0.544 [0.205, 0.729]
Left pedal cycle-averaged vertical force	15	Gear ratio	None	708.891	1.000	14.000	2.158870e-13	3.238305e-12	0.981 [0.952, 0.990]
Left pedal cycle-averaged vertical force	15	Posture × cadence	None	0.575	4.000	56.000	0.681958	1.000000	0.039 [0.000, 0.118]
Left pedal cycle-averaged vertical force	15	Posture × gear ratio	None	0.431	2.000	28.000	0.653818	1.000000	0.030 [0.000, 0.189]
Left pedal cycle-averaged vertical force	15	Cadence × gear ratio	GG	2.564	1.307	18.295	0.119832	0.789501	0.155 [0.000, 0.441]
Left pedal cycle-averaged vertical force	15	Posture × cadence × gear ratio	None	0.583	4.000	56.000	0.676198	1.000000	0.040 [0.000, 0.119]
Right pedal cycle-averaged vertical force	15	Posture	None	2.515	2.000	28.000	0.098997	0.821459	0.152 [0.000, 0.376]
Right pedal cycle-averaged vertical force	15	Cadence	None	20.386	2.000	28.000	3.438910e-06	2.751128e-05	0.593 [0.324, 0.737]
Right pedal cycle-averaged vertical force	15	Gear ratio	None	492.083	1.000	14.000	2.629030e-12	3.680642e-11	0.972 [0.932, 0.985]
Right pedal cycle-averaged vertical force	15	Posture × cadence	GG	0.947	2.348	32.869	0.410811	1.000000	0.063 [0.000, 0.233]
Right pedal cycle-averaged vertical force	15	Posture × gear ratio	None	0.691	2.000	28.000	0.509609	1.000000	0.047 [0.000, 0.227]
Right pedal cycle-averaged vertical force	15	Cadence × gear ratio	None	4.550	2.000	28.000	0.019460	0.194595	0.245 [0.006, 0.470]
Right pedal cycle-averaged vertical force	15	Posture × cadence × gear ratio	None	0.593	4.000	56.000	0.669215	1.000000	0.041 [0.000, 0.121]
Saddle cycle-averaged vertical force	20	Posture	None	0.870	2.000	38.000	0.427027	1.000000	0.044 [0.000, 0.196]
Saddle cycle-averaged vertical force	20	Cadence	None	0.024	2.000	38.000	0.976675	1.000000	0.001 [0.000, 0.000]
Saddle cycle-averaged vertical force	20	Gear ratio	None	102.266	1.000	19.000	4.392520e-09	3.953268e-08	0.843 [0.678, 0.910]
Saddle cycle-averaged vertical force	20	Posture × cadence	GG	0.747	2.091	39.722	0.485895	1.000000	0.038 [0.000, 0.178]
Saddle cycle-averaged vertical force	20	Posture × gear ratio	None	0.180	2.000	38.000	0.835678	1.000000	0.009 [0.000, 0.098]
Saddle cycle-averaged vertical force	20	Cadence × gear ratio	None	1.833	2.000	38.000	0.173734	0.789501	0.088 [0.000, 0.267]
Saddle cycle-averaged vertical force	20	Posture × cadence × gear ratio	None	0.471	4.000	76.000	0.757140	1.000000	0.024 [0.000, 0.076]

Note: Corr. indicates the sphericity correction applied to the reported degrees of freedom and p value.

Table S6. Post hoc paired comparisons for significant posture main effects on the original measurement scale.

Outcome	Comparison	Mean 1	Mean 2	Mean difference [95% CI]	t (df)	F (df1, df2)	praw	pHolm	ηp² [95% CI]
Left grip peak force	Comfort vs neutral	1.077	1.146	-0.069 [-0.091, -0.047]	-6.484 (19)	42.043 (1, 19)	3.262275e-06	9.786824e-06	0.689 [0.407, 0.819]
Left grip peak force	Comfort vs athletic	1.077	1.142	-0.065 [-0.094, -0.035]	-4.546 (19)	20.666 (1, 19)	0.000221	0.000442	0.521 [0.185, 0.717]
Left grip peak force	Neutral vs athletic	1.146	1.142	0.004 [-0.020, 0.029]	0.368 (19)	0.135 (1, 19)	0.717028	0.717028	0.007 [0.000, 0.211]
Left grip impulse per cycle	Comfort vs neutral	0.407	0.440	-0.033 [-0.046, -0.021]	-5.468 (19)	29.901 (1, 19)	2.824907e-05	8.474720e-05	0.611 [0.294, 0.773]
Left grip impulse per cycle	Comfort vs athletic	0.407	0.432	-0.025 [-0.040, -0.010]	-3.604 (19)	12.990 (1, 19)	0.001890	0.003781	0.406 [0.082, 0.642]
Left grip impulse per cycle	Neutral vs athletic	0.440	0.432	0.008 [-0.003, 0.020]	1.569 (19)	2.463 (1, 19)	0.133090	0.133090	0.115 [0.000, 0.402]
Right grip peak force	Comfort vs neutral	0.970	1.028	-0.058 [-0.074, -0.042]	-7.442 (19)	55.385 (1, 19)	4.820637e-07	1.446191e-06	0.745 [0.498, 0.852]

Outcome	Comparison	Mean 1	Mean 2	Mean difference [95% CI]	t (df)	F (df1, df2)	praw	pHolm	ηp² [95% CI]
Right grip peak force	Comfort vs athletic	0.970	1.041	-0.070 [-0.093, -0.048]	-6.647 (19)	44.178 (1, 19)	2.337874e-06	4.675748e-06	0.699 [0.423, 0.825]
Right grip peak force	Neutral vs athletic	1.028	1.041	-0.012 [-0.041, 0.016]	-0.909 (19)	0.825 (1, 19)	0.374979	0.374979	0.042 [0.000, 0.303]
Right grip impulse per cycle	Comfort vs neutral	0.352	0.374	-0.022 [-0.033, -0.012]	-4.615 (19)	21.294 (1, 19)	0.000189	0.000567	0.528 [0.193, 0.722]
Right grip impulse per cycle	Comfort vs athletic	0.352	0.375	-0.023 [-0.035, -0.012]	-4.180 (19)	17.473 (1, 19)	0.000508	0.001016	0.479 [0.143, 0.690]
Right grip impulse per cycle	Neutral vs athletic	0.374	0.375	-0.001 [-0.015, 0.013]	-0.125 (19)	0.016 (1, 19)	0.902207	0.902207	0.001 [0.000, 0.126]
Left grip cycle-averaged vertical force	Comfort vs neutral	0.490	0.529	-0.038 [-0.053, -0.024]	-5.559 (19)	30.906 (1, 19)	2.315670e-05	6.947010e-05	0.619 [0.305, 0.778]
Left grip cycle-averaged vertical force	Comfort vs athletic	0.490	0.519	-0.028 [-0.045, -0.012]	-3.621 (19)	13.111 (1, 19)	0.001820	0.003640	0.408 [0.084, 0.643]
Left grip cycle-averaged vertical force	Neutral vs athletic	0.529	0.519	0.010 [-0.004, 0.024]	1.464 (19)	2.143 (1, 19)	0.159575	0.159575	0.101 [0.000, 0.386]
Right grip cycle-averaged vertical force	Comfort vs neutral	0.431	0.458	-0.027 [-0.038, -0.016]	-4.971 (19)	24.706 (1, 19)	8.491230e-05	0.000255	0.565 [0.236, 0.745]
Right grip cycle-averaged vertical force	Comfort vs athletic	0.431	0.459	-0.028 [-0.040, -0.015]	-4.706 (19)	22.146 (1, 19)	0.000154	0.000308	0.538 [0.204, 0.728]
Right grip cycle-averaged vertical force	Neutral vs athletic	0.458	0.459	-0.001 [-0.017, 0.015]	-0.136 (19)	0.018 (1, 19)	0.893490	0.893490	0.001 [0.000, 0.133]

Table S7. Post hoc paired comparisons for significant cadence main effects on the original measurement scale.

Outcome	Comparison	Mean 1	Mean 2	Mean difference [95% CI]	t (df)	F (df1, df2)	praw	pHolm	ηp² [95% CI]
Left grip impulse per cycle	Slow vs self-selected	0.488	0.421	0.066 [0.042, 0.090]	5.847 (19)	34.184 (1, 19)	1.245080e-05	1.245080e-05	0.643 [0.338, 0.792]
Left grip impulse per cycle	Slow vs quick	0.488	0.370	0.118 [0.084, 0.152]	7.239 (19)	52.401 (1, 19)	7.157919e-07	1.431584e-06	0.734 [0.480, 0.846]
Left grip impulse per cycle	Self-selected vs quick	0.421	0.370	0.052 [0.039, 0.065]	8.202 (19)	67.279 (1, 19)	1.154136e-07	3.462407e-07	0.780 [0.560, 0.873]
Right grip impulse per cycle	Slow vs self-selected	0.420	0.365	0.055 [0.047, 0.063]	13.688 (19)	187.374 (1, 19)	2.720784e-11	5.441569e-11	0.908 [0.807, 0.947]
Right grip impulse per cycle	Slow vs quick	0.420	0.317	0.103 [0.088, 0.118]	14.465 (19)	209.242 (1, 19)	1.040549e-11	3.121648e-11	0.917 [0.825, 0.952]
Right grip impulse per cycle	Self-selected vs quick	0.365	0.317	0.048 [0.039, 0.057]	11.383 (19)	129.572 (1, 19)	6.285589e-10	6.285589e-10	0.872 [0.735, 0.926]
Left pedal peak force	Slow vs self-selected	3.391	3.604	-0.213 [-0.273, -0.153]	-7.539 (15)	56.832 (1, 15)	1.775413e-06	3.550826e-06	0.791 [0.538, 0.886]
Left pedal peak force	Slow vs quick	3.391	3.860	-0.469 [-0.559, -0.380]	-11.147 (15)	124.266 (1, 15)	1.175481e-08	3.526443e-08	0.892 [0.750, 0.941]
Left pedal peak force	Self-selected vs quick	3.604	3.860	-0.256 [-0.371, -0.141]	-4.752 (15)	22.582 (1, 15)	0.000257	0.000257	0.601 [0.231, 0.780]
Left pedal impulse per cycle	Slow vs self-selected	1.150	1.025	0.125 [0.094, 0.155]	8.769 (14)	76.892 (1, 14)	4.632679e-07	1.389804e-06	0.846 [0.638, 0.918]
Left pedal impulse per cycle	Slow vs quick	1.150	0.929	0.221 [0.160, 0.281]	7.798 (14)	60.801 (1, 14)	1.843313e-06	3.686626e-06	0.813 [0.568, 0.900]
Left pedal impulse per cycle	Self-selected vs quick	1.025	0.929	0.096 [0.052, 0.140]	4.638 (14)	21.508 (1, 14)	0.000384	0.000384	0.606 [0.221, 0.787]
Right pedal peak force	Slow vs self-selected	3.358	3.637	-0.279 [-0.366, -0.192]	-6.802 (15)	46.265 (1, 15)	5.974283e-06	1.194857e-05	0.755 [0.470, 0.866]
Right pedal peak force	Slow vs quick	3.358	3.761	-0.403 [-0.514, -0.292]	-7.723 (15)	59.652 (1, 15)	1.324401e-06	3.973202e-06	0.799 [0.554, 0.891]
Right pedal peak force	Self-selected vs quick	3.637	3.761	-0.124 [-0.194, -0.054]	-3.771 (15)	14.218 (1, 15)	0.001851	0.001851	0.487 [0.109, 0.713]
Right pedal impulse per cycle	Slow vs self-selected	1.148	1.031	0.117 [0.072, 0.162]	5.586 (14)	31.208 (1, 14)	6.706769e-05	6.706769e-05	0.690 [0.343, 0.834]
Right pedal impulse per cycle	Slow vs quick	1.148	0.928	0.220 [0.159, 0.280]	7.814 (14)	61.055 (1, 14)	1.799483e-06	3.598965e-06	0.813 [0.569, 0.900]
Right pedal impulse per cycle	Self-selected vs quick	1.031	0.928	0.103 [0.076, 0.129]	8.296 (14)	68.828 (1, 14)	8.945705e-07	2.683712e-06	0.831 [0.606, 0.910]

Outcome	Comparison	Mean 1	Mean 2	Mean difference [95% CI]	t (df)	F (df1, df2)	praw	pHolm	ηp² [95% CI]
Saddle impulse per cycle	Slow vs self-selected	1.888	1.619	0.269 [0.190, 0.348]	7.106 (19)	50.493 (1, 19)	9.299429e-07	1.859886e-06	0.727 [0.468, 0.841]
Saddle impulse per cycle	Slow vs quick	1.888	1.423	0.465 [0.334, 0.595]	7.470 (19)	55.801 (1, 19)	4.568167e-07	1.370450e-06	0.746 [0.501, 0.853]
Saddle impulse per cycle	Self-selected vs quick	1.619	1.423	0.195 [0.129, 0.262]	6.180 (19)	38.191 (1, 19)	6.142228e-06	6.142228e-06	0.668 [0.375, 0.807]
Left pedal cycle-averaged vertical force	Slow vs self-selected	1.198	1.241	-0.043 [-0.065, -0.021]	-4.282 (14)	18.335 (1, 14)	0.000760	0.001519	0.567 [0.175, 0.765]
Left pedal cycle-averaged vertical force	Slow vs quick	1.198	1.285	-0.086 [-0.118, -0.055]	-5.959 (14)	35.504 (1, 14)	3.495730e-05	0.000105	0.717 [0.388, 0.848]
Left pedal cycle-averaged vertical force	Self-selected vs quick	1.241	1.285	-0.043 [-0.084, -0.003]	-2.291 (14)	5.251 (1, 14)	0.037961	0.037961	0.273 [0.000, 0.579]
Right pedal cycle-averaged vertical force	Slow vs self-selected	1.195	1.251	-0.057 [-0.080, -0.033]	-5.088 (14)	25.883 (1, 14)	0.000165	0.000331	0.649 [0.280, 0.811]
Right pedal cycle-averaged vertical force	Slow vs quick	1.195	1.282	-0.087 [-0.120, -0.055]	-5.792 (14)	33.545 (1, 14)	4.669890e-05	0.000140	0.706 [0.368, 0.842]
Right pedal cycle-averaged vertical force	Self-selected vs quick	1.251	1.282	-0.031 [-0.063, 0.002]	-2.044 (14)	4.179 (1, 14)	0.060213	0.060213	0.230 [0.000, 0.547]

Table S8. Heavy-versus-light gear ratio comparisons on the original measurement scale.

Outcome	Comparison	Mean 1	Mean 2	Mean difference [95% CI]	t (df)	F (df1, df2)	praw	pHolm	ηp² [95% CI]
Left grip peak force	Heavy vs light	1.091	1.153	-0.062 [-0.086, -0.037]	-5.276 (19)	27.839 (1, 19)	4.305531e-05	8.611062e-05	0.594 [0.272, 0.762]
Left grip impulse per cycle	Heavy vs light	0.402	0.451	-0.049 [-0.062, -0.035]	-7.582 (19)	57.492 (1, 19)	3.681664e-07	1.104499e-06	0.752 [0.510, 0.856]
Right grip peak force	Heavy vs light	0.978	1.048	-0.070 [-0.098, -0.042]	-5.262 (19)	27.689 (1, 19)	4.442977e-05	8.611062e-05	0.593 [0.270, 0.762]
Right grip impulse per cycle	Heavy vs light	0.340	0.394	-0.054 [-0.068, -0.041]	-8.244 (19)	67.967 (1, 19)	1.069157e-07	5.270921e-07	0.782 [0.563, 0.874]
Left pedal peak force	Heavy vs light	4.388	2.849	1.539 [1.353, 1.726]	17.570 (15)	308.688 (1, 15)	2.043954e-11	2.452745e-10	0.954 [0.890, 0.975]
Left pedal impulse per cycle	Heavy vs light	1.296	0.773	0.523 [0.457, 0.590]	16.778 (14)	281.488 (1, 14)	1.146673e-10	1.261340e-09	0.953 [0.883, 0.975]
Right pedal peak force	Heavy vs light	4.372	2.799	1.573 [1.383, 1.762]	17.699 (15)	313.237 (1, 15)	1.840256e-11	2.392332e-10	0.954 [0.892, 0.975]
Right pedal impulse per cycle	Heavy vs light	1.288	0.784	0.504 [0.436, 0.572]	15.869 (14)	251.821 (1, 14)	2.410745e-10	2.410745e-09	0.947 [0.871, 0.972]
Saddle peak force	Heavy vs light	3.904	4.723	-0.819 [-1.024, -0.614]	-8.352 (19)	69.761 (1, 19)	8.784868e-08	5.270921e-07	0.786 [0.571, 0.876]
Saddle impulse per cycle	Heavy vs light	1.444	1.843	-0.399 [-0.487, -0.311]	-9.462 (19)	89.523 (1, 19)	1.273690e-08	1.018952e-07	0.825 [0.643, 0.899]
Left grip cycle-averaged vertical force	Heavy vs light	0.483	0.542	-0.059 [-0.074, -0.045]	-8.483 (19)	71.963 (1, 19)	6.940050e-08	4.858035e-07	0.791 [0.580, 0.879]
Right grip cycle-averaged vertical force	Heavy vs light	0.415	0.484	-0.069 [-0.087, -0.052]	-8.276 (19)	68.484 (1, 19)	1.009890e-07	5.270921e-07	0.783 [0.565, 0.874]
Left pedal cycle-averaged vertical force	Heavy vs light	1.556	0.927	0.629 [0.579, 0.680]	26.625 (14)	708.891 (1, 14)	2.158870e-13	3.238305e-12	0.981 [0.952, 0.990]
Right pedal cycle-averaged vertical force	Heavy vs light	1.546	0.939	0.607 [0.548, 0.666]	22.183 (14)	492.083 (1, 14)	2.629030e-12	3.680642e-11	0.972 [0.932, 0.985]
Saddle cycle-averaged vertical force	Heavy vs light	1.715	2.205	-0.490 [-0.591, -0.389]	-10.113 (19)	102.266 (1, 19)	4.392520e-09	3.953268e-08	0.843 [0.678, 0.910]

Note: Mean 1 corresponds to heavy gear ratio and Mean 2 to light gear ratio. For this two-level main effect, pHolm is the 15-outcome Holm-adjusted omnibus p value.

Table S9. Cadence simple effects within each gear ratio level for significant original-scale cadence × gear ratio interactions.

Outcome	Gear ratio	Comparison	Mean 1	Mean 2	Mean difference [95% CI]	t (df)	F (df1, df2)	praw	pHolm	ηp² [95% CI]
Left pedal impulse per cycle	Light	Slow vs self-selected	0.862	0.766	0.096 [0.074, 0.118]	9.310 (14)	86.672 (1, 14)	2.248454e-07	1.349072e-06	0.861 [0.670, 0.926]
Left pedal impulse per cycle	Light	Slow vs quick	0.862	0.691	0.171 [0.108, 0.235]	5.803 (14)	33.675 (1, 14)	4.578783e-05	9.157567e-05	0.706 [0.369, 0.842]
Left pedal impulse per cycle	Light	Self-selected vs quick	0.766	0.691	0.075 [0.016, 0.135]	2.712 (14)	7.353 (1, 14)	0.016865	0.016865	0.344 [0.012, 0.629]
Left pedal impulse per cycle	Heavy	Slow vs self-selected	1.437	1.284	0.153 [0.109, 0.198]	7.455 (14)	55.575 (1, 14)	3.083436e-06	1.233374e-05	0.799 [0.539, 0.892]
Left pedal impulse per cycle	Heavy	Slow vs quick	1.437	1.167	0.270 [0.202, 0.337]	8.576 (14)	73.554 (1, 14)	6.038090e-07	3.019045e-06	0.840 [0.625, 0.915]
Left pedal impulse per cycle	Heavy	Self-selected vs quick	1.284	1.167	0.117 [0.080, 0.153]	6.874 (14)	47.245 (1, 14)	7.640864e-06	2.292259e-05	0.771 [0.486, 0.878]
Right pedal impulse per cycle	Light	Slow vs self-selected	0.873	0.785	0.088 [0.049, 0.127]	4.806 (14)	23.097 (1, 14)	0.000279	0.000279	0.623 [0.243, 0.796]
Right pedal impulse per cycle	Light	Slow vs quick	0.873	0.694	0.178 [0.125, 0.232]	7.131 (14)	50.851 (1, 14)	5.083880e-06	2.033552e-05	0.784 [0.510, 0.884]
Right pedal impulse per cycle	Light	Self-selected vs quick	0.785	0.694	0.090 [0.072, 0.109]	10.416 (14)	108.498 (1, 14)	5.623825e-08	3.374295e-07	0.886 [0.726, 0.939]
Right pedal impulse per cycle	Heavy	Slow vs self-selected	1.424	1.277	0.147 [0.089, 0.204]	5.502 (14)	30.274 (1, 14)	7.791911e-05	0.000173	0.684 [0.333, 0.830]
Right pedal impulse per cycle	Heavy	Slow vs quick	1.424	1.162	0.261 [0.191, 0.332]	7.956 (14)	63.299 (1, 14)	1.460007e-06	7.300037e-06	0.819 [0.580, 0.903]
Right pedal impulse per cycle	Heavy	Self-selected vs quick	1.277	1.162	0.115 [0.071, 0.158]	5.672 (14)	32.168 (1, 14)	5.766487e-05	0.000173	0.697 [0.353, 0.837]
Right grip cycle-averaged vertical force	Light	Slow vs self-selected	0.472	0.492	-0.019 [-0.030, -0.008]	-3.580 (19)	12.814 (1, 19)	0.001999	0.010604	0.403 [0.080, 0.640]
Right grip cycle-averaged vertical force	Light	Slow vs quick	0.472	0.489	-0.016 [-0.029, -0.003]	-2.663 (19)	7.093 (1, 19)	0.015354	0.046062	0.272 [0.011, 0.545]
Right grip cycle-averaged vertical force	Light	Self-selected vs quick	0.492	0.489	0.003 [-0.009, 0.014]	0.512 (19)	0.262 (1, 19)	0.614615	0.614615	0.014 [0.000, 0.239]
Right grip cycle-averaged vertical force	Heavy	Slow vs self-selected	0.427	0.413	0.014 [0.006, 0.021]	3.634 (19)	13.204 (1, 19)	0.001767	0.010604	0.410 [0.085, 0.645]
Right grip cycle-averaged vertical force	Heavy	Slow vs quick	0.427	0.404	0.023 [0.009, 0.036]	3.476 (19)	12.080 (1, 19)	0.002531	0.010604	0.389 [0.070, 0.630]
Right grip cycle-averaged vertical force	Heavy	Self-selected vs quick	0.413	0.404	0.009 [-0.003, 0.021]	1.547 (19)	2.392 (1, 19)	0.138453	0.276907	0.112 [0.000, 0.398]

Table S10. Gear ratio simple effects within each cadence level for significant original-scale cadence × gear ratio interactions.

Outcome	Cadence	Comparison	Mean 1	Mean 2	Mean difference [95% CI]	t (df)	F (df1, df2)	praw	pHolm	ηp² [95% CI]
Left pedal impulse per cycle	Slow	Heavy vs light	1.437	0.862	0.575 [0.494, 0.657]	15.153 (14)	229.619 (1, 14)	4.448955e-10	8.897909e-10	0.943 [0.859, 0.969]
Left pedal impulse per cycle	Self-selected	Heavy vs light	1.284	0.766	0.518 [0.459, 0.578]	18.665 (14)	348.388 (1, 14)	2.737039e-11	8.211116e-11	0.961 [0.905, 0.979]
Left pedal impulse per cycle	Quick	Heavy vs light	1.167	0.691	0.477 [0.406, 0.548]	14.379 (14)	206.761 (1, 14)	8.891574e-10	8.897909e-10	0.937 [0.845, 0.966]
Right pedal impulse per cycle	Slow	Heavy vs light	1.424	0.873	0.551 [0.477, 0.625]	15.928 (14)	253.713 (1, 14)	2.293591e-10	6.880774e-10	0.948 [0.872, 0.972]
Right pedal impulse per cycle	Self-selected	Heavy vs light	1.277	0.785	0.492 [0.416, 0.568]	13.812 (14)	190.768 (1, 14)	1.508679e-09	1.508679e-09	0.932 [0.833, 0.963]
Right pedal impulse per cycle	Quick	Heavy vs light	1.162	0.694	0.468 [0.405, 0.531]	15.910 (14)	253.124 (1, 14)	2.329315e-10	6.880774e-10	0.948 [0.871, 0.972]
Right grip cycle-averaged vertical force	Slow	Heavy vs light	0.427	0.472	-0.046 [-0.066, -0.025]	-4.683 (19)	21.928 (1, 19)	0.000162	0.000162	0.536 [0.201, 0.726]

Outcome	Cadence	Comparison	Mean 1	Mean 2	Mean difference [95% CI]	t (df)	F (df1, df2)	praw	pHolm	ηp² [95% CI]
Right grip cycle-averaged vertical force	Self-selected	Heavy vs light	0.413	0.492	-0.078 [-0.098, -0.058]	-8.267 (19)	68.348 (1, 19)	1.025140e-07	3.075430e-07	0.782 [0.565, 0.874]
Right grip cycle-averaged vertical force	Quick	Heavy vs light	0.404	0.489	-0.085 [-0.106, -0.063]	-8.139 (19)	66.249 (1, 19)	1.295590e-07	3.075430e-07	0.777 [0.555, 0.871]

Note: Mean 1 corresponds to heavy gear ratio and Mean 2 to light gear ratio.

Table S11. Complete 3 × 3 × 2 repeated-measures ANOVA results after natural-log transformation (sensitivity analysis).

Outcome	n	Effect	Corr.	F	df1	df2	p	pHolm	ηp² [95% CI]
Left grip peak force	20	Posture	None	17.468	2.000	38.000	4.169600e-06	6.254400e-05	0.479 [0.233, 0.638]
Left grip peak force	20	Cadence	None	0.280	2.000	38.000	0.757154	1.000000	0.015 [0.000, 0.121]
Left grip peak force	20	Gear ratio	None	23.057	1.000	19.000	0.000124	0.000124	0.548 [0.215, 0.734]
Left grip peak force	20	Posture × cadence	None	1.765	4.000	76.000	0.144754	1.000000	0.085 [0.000, 0.189]
Left grip peak force	20	Posture × gear ratio	None	1.864	2.000	38.000	0.169026	1.000000	0.089 [0.000, 0.269]
Left grip peak force	20	Cadence × gear ratio	None	2.152	2.000	38.000	0.130243	1.000000	0.102 [0.000, 0.286]
Left grip peak force	20	Posture × cadence × gear ratio	None	0.268	4.000	76.000	0.897374	1.000000	0.014 [0.000, 0.043]
Left grip impulse per cycle	20	Posture	None	14.314	2.000	38.000	2.325096e-05	0.000326	0.430 [0.180, 0.600]
Left grip impulse per cycle	20	Cadence	None	205.524	2.000	38.000	4.191032e-21	6.286548e-20	0.915 [0.860, 0.943]
Left grip impulse per cycle	20	Gear ratio	None	77.262	1.000	19.000	4.027046e-08	3.624341e-07	0.803 [0.601, 0.886]
Left grip impulse per cycle	20	Posture × cadence	None	2.244	4.000	76.000	0.072074	1.000000	0.106 [0.000, 0.218]
Left grip impulse per cycle	20	Posture × gear ratio	GG	0.803	1.484	28.194	0.423951	1.000000	0.041 [0.000, 0.233]
Left grip impulse per cycle	20	Cadence × gear ratio	None	2.996	2.000	38.000	0.061908	0.804808	0.136 [0.000, 0.329]
Left grip impulse per cycle	20	Posture × cadence × gear ratio	None	0.801	4.000	76.000	0.528533	1.000000	0.040 [0.000, 0.114]
Right grip peak force	20	Posture	GG	18.518	1.463	27.789	3.646205e-05	0.000438	0.494 [0.210, 0.671]
Right grip peak force	20	Cadence	None	1.250	2.000	38.000	0.298096	1.000000	0.062 [0.000, 0.227]
Right grip peak force	20	Gear ratio	None	27.084	1.000	19.000	5.048181e-05	0.000101	0.588 [0.263, 0.758]
Right grip peak force	20	Posture × cadence	GG	0.394	2.274	43.215	0.702734	1.000000	0.020 [0.000, 0.124]
Right grip peak force	20	Posture × gear ratio	GG	1.155	1.481	28.135	0.315184	1.000000	0.057 [0.000, 0.263]
Right grip peak force	20	Cadence × gear ratio	None	2.191	2.000	38.000	0.125738	1.000000	0.103 [0.000, 0.288]
Right grip peak force	20	Posture × cadence × gear ratio	None	0.649	4.000	76.000	0.629181	1.000000	0.033 [0.000, 0.098]
Right grip impulse per cycle	20	Posture	None	8.924	2.000	38.000	0.000665	0.007314	0.320 [0.081, 0.510]
Right grip impulse per cycle	20	Cadence	GG	491.430	1.414	26.858	2.332771e-20	3.265880e-19	0.963 [0.931, 0.977]
Right grip impulse per cycle	20	Gear ratio	None	69.225	1.000	19.000	9.312085e-08	6.518459e-07	0.785 [0.569, 0.875]
Right grip impulse per cycle	20	Posture × cadence	None	0.176	4.000	76.000	0.950231	1.000000	0.009 [0.000, 0.020]
Right grip impulse per cycle	20	Posture × gear ratio	None	0.525	2.000	38.000	0.595850	1.000000	0.027 [0.000, 0.159]

Outcome	n	Effect	Corr.	F	df1	df2	p	pHolm	ηp² [95% CI]
Right grip impulse per cycle	20	Cadence × gear ratio	None	11.101	2.000	38.000	0.000160	0.002396	0.369 [0.122, 0.551]
Right grip impulse per cycle	20	Posture × cadence × gear ratio	None	0.837	4.000	76.000	0.505642	1.000000	0.042 [0.000, 0.117]
Left pedal peak force	16	Posture	None	0.389	2.000	30.000	0.681257	1.000000	0.025 [0.000, 0.171]
Left pedal peak force	16	Cadence	None	72.953	2.000	30.000	3.003522e-12	3.303875e-11	0.829 [0.700, 0.891]
Left pedal peak force	16	Gear ratio	None	422.364	1.000	15.000	2.126648e-12	2.126648e-11	0.966 [0.918, 0.981]
Left pedal peak force	16	Posture × cadence	GG	0.605	2.295	34.427	0.573835	1.000000	0.039 [0.000, 0.183]
Left pedal peak force	16	Posture × gear ratio	None	0.190	2.000	30.000	0.828059	1.000000	0.012 [0.000, 0.124]
Left pedal peak force	16	Cadence × gear ratio	None	1.022	2.000	30.000	0.371984	1.000000	0.064 [0.000, 0.252]
Left pedal peak force	16	Posture × cadence × gear ratio	None	1.500	4.000	60.000	0.213518	1.000000	0.091 [0.000, 0.208]
Left pedal impulse per cycle	15	Posture	None	2.058	2.000	28.000	0.146542	1.000000	0.128 [0.000, 0.348]
Left pedal impulse per cycle	15	Cadence	GG	104.666	1.321	18.497	9.204749e-10	9.204749e-09	0.882 [0.751, 0.932]
Left pedal impulse per cycle	15	Gear ratio	None	1866.297	1.000	14.000	2.665987e-16	3.998981e-15	0.993 [0.982, 0.996]
Left pedal impulse per cycle	15	Posture × cadence	None	0.453	4.000	56.000	0.769554	1.000000	0.031 [0.000, 0.098]
Left pedal impulse per cycle	15	Posture × gear ratio	None	0.555	2.000	28.000	0.580383	1.000000	0.038 [0.000, 0.209]
Left pedal impulse per cycle	15	Cadence × gear ratio	GG	0.195	1.296	18.138	0.727394	1.000000	0.014 [0.000, 0.222]
Left pedal impulse per cycle	15	Posture × cadence × gear ratio	None	0.690	4.000	56.000	0.601981	1.000000	0.047 [0.000, 0.134]
Right pedal peak force	16	Posture	None	1.130	2.000	30.000	0.336535	1.000000	0.070 [0.000, 0.262]
Right pedal peak force	16	Cadence	GG	50.641	1.413	21.196	6.616540e-08	5.954886e-07	0.771 [0.559, 0.864]
Right pedal peak force	16	Gear ratio	None	494.294	1.000	15.000	6.773287e-13	7.450616e-12	0.971 [0.930, 0.984]
Right pedal peak force	16	Posture × cadence	GG	0.467	2.263	33.944	0.654603	1.000000	0.030 [0.000, 0.165]
Right pedal peak force	16	Posture × gear ratio	None	0.774	2.000	30.000	0.470100	1.000000	0.049 [0.000, 0.225]
Right pedal peak force	16	Cadence × gear ratio	None	0.445	2.000	30.000	0.644737	1.000000	0.029 [0.000, 0.181]
Right pedal peak force	16	Posture × cadence × gear ratio	GG	0.180	2.196	32.946	0.855099	1.000000	0.012 [0.000, 0.105]
Right pedal impulse per cycle	15	Posture	GG	2.161	1.430	20.025	0.151464	1.000000	0.134 [0.000, 0.404]
Right pedal impulse per cycle	15	Cadence	None	121.115	2.000	28.000	1.644148e-14	1.972978e-13	0.896 [0.812, 0.935]
Right pedal impulse per cycle	15	Gear ratio	None	819.882	1.000	14.000	7.934215e-14	1.031448e-12	0.983 [0.958, 0.991]
Right pedal impulse per cycle	15	Posture × cadence	GG	1.152	1.809	25.327	0.327462	1.000000	0.076 [0.000, 0.293]
Right pedal impulse per cycle	15	Posture × gear ratio	None	0.706	2.000	28.000	0.502427	1.000000	0.048 [0.000, 0.229]
Right pedal impulse per cycle	15	Cadence × gear ratio	None	1.573	2.000	28.000	0.225183	1.000000	0.101 [0.000, 0.314]
Right pedal impulse per cycle	15	Posture × cadence × gear ratio	None	0.682	4.000	56.000	0.607276	1.000000	0.046 [0.000, 0.133]
Saddle peak force	20	Posture	None	0.149	2.000	38.000	0.861905	1.000000	0.008 [0.000, 0.089]
Saddle peak force	20	Cadence	None	0.270	2.000	38.000	0.764535	1.000000	0.014 [0.000, 0.119]

Outcome	n	Effect	Corr.	F	df1	df2	p	pHolm	ηp² [95% CI]
Saddle peak force	20	Gear ratio	None	34.744	1.000	19.000	1.124347e-05	3.373042e-05	0.646 [0.343, 0.794]
Saddle peak force	20	Posture × cadence	GG	1.160	1.884	35.795	0.322467	1.000000	0.058 [0.000, 0.228]
Saddle peak force	20	Posture × gear ratio	GG	0.138	1.394	26.482	0.795036	1.000000	0.007 [0.000, 0.144]
Saddle peak force	20	Cadence × gear ratio	None	1.154	2.000	38.000	0.326203	1.000000	0.057 [0.000, 0.220]
Saddle peak force	20	Posture × cadence × gear ratio	GG	0.665	2.270	43.124	0.537582	1.000000	0.034 [0.000, 0.159]
Saddle impulse per cycle	20	Posture	GG	0.358	1.405	26.689	0.627259	1.000000	0.019 [0.000, 0.190]
Saddle impulse per cycle	20	Cadence	None	98.903	2.000	38.000	8.656521e-16	1.125348e-14	0.839 [0.736, 0.892]
Saddle impulse per cycle	20	Gear ratio	None	49.151	1.000	19.000	1.122863e-06	5.614314e-06	0.721 [0.459, 0.838]
Saddle impulse per cycle	20	Posture × cadence	GG	0.454	1.773	33.690	0.615572	1.000000	0.023 [0.000, 0.166]
Saddle impulse per cycle	20	Posture × gear ratio	None	0.293	2.000	38.000	0.747507	1.000000	0.015 [0.000, 0.124]
Saddle impulse per cycle	20	Cadence × gear ratio	None	0.577	2.000	38.000	0.566181	1.000000	0.029 [0.000, 0.165]
Saddle impulse per cycle	20	Posture × cadence × gear ratio	GG	0.290	2.332	44.313	0.782125	1.000000	0.015 [0.000, 0.103]
Left grip cycle-averaged vertical force	20	Posture	None	14.314	2.000	38.000	2.325096e-05	0.000326	0.430 [0.180, 0.600]
Left grip cycle-averaged vertical force	20	Cadence	None	0.279	2.000	38.000	0.758366	1.000000	0.014 [0.000, 0.121]
Left grip cycle-averaged vertical force	20	Gear ratio	None	77.262	1.000	19.000	4.027046e-08	3.624341e-07	0.803 [0.601, 0.886]
Left grip cycle-averaged vertical force	20	Posture × cadence	None	2.244	4.000	76.000	0.072074	1.000000	0.106 [0.000, 0.218]
Left grip cycle-averaged vertical force	20	Posture × gear ratio	GG	0.803	1.484	28.194	0.423951	1.000000	0.041 [0.000, 0.233]
Left grip cycle-averaged vertical force	20	Cadence × gear ratio	None	2.996	2.000	38.000	0.061908	0.804808	0.136 [0.000, 0.329]
Left grip cycle-averaged vertical force	20	Posture × cadence × gear ratio	None	0.801	4.000	76.000	0.528533	1.000000	0.040 [0.000, 0.114]
Right grip cycle-averaged vertical force	20	Posture	None	8.924	2.000	38.000	0.000665	0.007314	0.320 [0.081, 0.510]
Right grip cycle-averaged vertical force	20	Cadence	GG	2.184	1.494	28.383	0.141800	0.850798	0.103 [0.000, 0.327]
Right grip cycle-averaged vertical force	20	Gear ratio	None	69.225	1.000	19.000	9.312085e-08	6.518459e-07	0.785 [0.569, 0.875]
Right grip cycle-averaged vertical force	20	Posture × cadence	None	0.176	4.000	76.000	0.950231	1.000000	0.009 [0.000, 0.020]
Right grip cycle-averaged vertical force	20	Posture × gear ratio	None	0.525	2.000	38.000	0.595850	1.000000	0.027 [0.000, 0.159]
Right grip cycle-averaged vertical force	20	Cadence × gear ratio	None	11.101	2.000	38.000	0.000160	0.002396	0.369 [0.122, 0.551]
Right grip cycle-averaged vertical force	20	Posture × cadence × gear ratio	None	0.837	4.000	76.000	0.505642	1.000000	0.042 [0.000, 0.117]
Left pedal cycle-averaged vertical force	15	Posture	None	2.058	2.000	28.000	0.146542	1.000000	0.128 [0.000, 0.348]
Left pedal cycle-averaged vertical force	15	Cadence	GG	15.293	1.361	19.054	0.000383	0.002679	0.522 [0.177, 0.716]
Left pedal cycle-averaged vertical force	15	Gear ratio	None	1866.297	1.000	14.000	2.665987e-16	3.998981e-15	0.993 [0.982, 0.996]
Left pedal cycle-averaged vertical force	15	Posture × cadence	None	0.453	4.000	56.000	0.769554	1.000000	0.031 [0.000, 0.098]
Left pedal cycle-averaged vertical force	15	Posture × gear ratio	None	0.555	2.000	28.000	0.580383	1.000000	0.038 [0.000, 0.209]
Left pedal cycle-averaged vertical force	15	Cadence × gear ratio	GG	0.195	1.296	18.138	0.727394	1.000000	0.014 [0.000, 0.222]

Outcome	n	Effect	Corr.	F	df1	df2	p	pHolm	ηp² [95% CI]
Left pedal cycle-averaged vertical force	15	Posture × cadence × gear ratio	None	0.690	4.000	56.000	0.601981	1.000000	0.047 [0.000, 0.134]
Right pedal cycle-averaged vertical force	15	Posture	GG	2.161	1.430	20.025	0.151464	1.000000	0.134 [0.000, 0.405]
Right pedal cycle-averaged vertical force	15	Cadence	None	22.894	2.000	28.000	1.283308e-06	1.026646e-05	0.621 [0.362, 0.756]
Right pedal cycle-averaged vertical force	15	Gear ratio	None	819.882	1.000	14.000	7.934215e-14	1.031448e-12	0.983 [0.958, 0.991]
Right pedal cycle-averaged vertical force	15	Posture × cadence	GG	1.152	1.809	25.327	0.327462	1.000000	0.076 [0.000, 0.293]
Right pedal cycle-averaged vertical force	15	Posture × gear ratio	None	0.706	2.000	28.000	0.502427	1.000000	0.048 [0.000, 0.229]
Right pedal cycle-averaged vertical force	15	Cadence × gear ratio	None	1.573	2.000	28.000	0.225183	1.000000	0.101 [0.000, 0.314]
Right pedal cycle-averaged vertical force	15	Posture × cadence × gear ratio	None	0.682	4.000	56.000	0.607276	1.000000	0.046 [0.000, 0.133]
Saddle cycle-averaged vertical force	20	Posture	GG	0.358	1.405	26.689	0.627259	1.000000	0.019 [0.000, 0.190]
Saddle cycle-averaged vertical force	20	Cadence	None	0.043	2.000	38.000	0.957565	1.000000	0.002 [0.000, 0.028]
Saddle cycle-averaged vertical force	20	Gear ratio	None	49.151	1.000	19.000	1.122863e-06	5.614314e-06	0.721 [0.459, 0.838]
Saddle cycle-averaged vertical force	20	Posture × cadence	GG	0.454	1.773	33.690	0.615572	1.000000	0.023 [0.000, 0.166]
Saddle cycle-averaged vertical force	20	Posture × gear ratio	None	0.293	2.000	38.000	0.747507	1.000000	0.015 [0.000, 0.124]
Saddle cycle-averaged vertical force	20	Cadence × gear ratio	None	0.577	2.000	38.000	0.566181	1.000000	0.029 [0.000, 0.165]
Saddle cycle-averaged vertical force	20	Posture × cadence × gear ratio	GG	0.290	2.332	44.313	0.782125	1.000000	0.015 [0.000, 0.103]

Note: Holm correction was applied across all 15 outcomes separately for each factorial effect, as in the primary analysis.

Table S12. Post hoc comparisons for significant posture and cadence main effects after natural-log transformation (sensitivity analysis).

Outcome	Main effect	Comparison	Mean 1	Mean 2	Mean difference [95% CI]	t (df)	F (df1, df2)	praw	pHolm	ηp² [95% CI]
Left grip peak force	Posture	Comfort vs neutral	0.048	0.112	-0.064 [-0.083, -0.044]	-6.758 (19)	45.669 (1, 19)	1.865096e-06	5.595288e-06	0.706 [0.434, 0.829]
Left grip peak force	Posture	Comfort vs athletic	0.048	0.110	-0.062 [-0.093, -0.031]	-4.148 (19)	17.202 (1, 19)	0.000547	0.001094	0.475 [0.139, 0.688]
Left grip peak force	Posture	Neutral vs athletic	0.112	0.110	0.002 [-0.023, 0.027]	0.171 (19)	0.029 (1, 19)	0.865878	0.865878	0.002 [0.000, 0.152]
Left grip impulse per cycle	Posture	Comfort vs neutral	-0.967	-0.885	-0.082 [-0.115, -0.049]	-5.220 (19)	27.246 (1, 19)	4.877655e-05	0.000146	0.589 [0.265, 0.759]
Left grip impulse per cycle	Posture	Comfort vs athletic	-0.967	-0.906	-0.061 [-0.098, -0.024]	-3.472 (19)	12.053 (1, 19)	0.002554	0.005108	0.388 [0.069, 0.630]
Left grip impulse per cycle	Posture	Neutral vs athletic	-0.885	-0.906	0.021 [-0.009, 0.051]	1.462 (19)	2.139 (1, 19)	0.159975	0.159975	0.101 [0.000, 0.386]
Right grip peak force	Posture	Comfort vs neutral	-0.044	0.016	-0.060 [-0.080, -0.040]	-6.318 (19)	39.915 (1, 19)	4.602750e-06	1.380825e-05	0.678 [0.389, 0.812]
Right grip peak force	Posture	Comfort vs athletic	-0.044	0.026	-0.069 [-0.093, -0.046]	-6.199 (19)	38.430 (1, 19)	5.898238e-06	1.380825e-05	0.669 [0.377, 0.807]
Right grip peak force	Posture	Neutral vs athletic	0.016	0.026	-0.009 [-0.042, 0.023]	-0.594 (19)	0.353 (1, 19)	0.559677	0.559677	0.018 [0.000, 0.253]
Right grip impulse per cycle	Posture	Comfort vs neutral	-1.072	-1.012	-0.060 [-0.090, -0.030]	-4.238 (19)	17.958 (1, 19)	0.000445	0.001336	0.486 [0.149, 0.695]
Right grip impulse per cycle	Posture	Comfort vs athletic	-1.072	-1.011	-0.061 [-0.092, -0.030]	-4.107 (19)	16.865 (1, 19)	0.000600	0.001336	0.470 [0.135, 0.684]
Right grip impulse per cycle	Posture	Neutral vs athletic	-1.012	-1.011	-0.001 [-0.042, 0.041]	-0.032 (19)	0.001 (1, 19)	0.974428	0.974428	0.000 [0.000, 0.002]

Outcome	Main effect	Comparison	Mean 1	Mean 2	Mean difference [95% CI]	t (df)	F (df1, df2)	praw	pHolm	ηp² [95% CI]
Left grip impulse per cycle	Cadence	Slow vs self-selected	-0.784	-0.921	0.138 [0.115, 0.160]	12.971 (19)	168.250 (1, 19)	6.885313e-11	1.377063e-10	0.899 [0.788, 0.941]
Left grip impulse per cycle	Cadence	Slow vs quick	-0.784	-1.054	0.270 [0.237, 0.303]	17.099 (19)	292.391 (1, 19)	5.382010e-13	1.614603e-12	0.939 [0.871, 0.965]
Left grip impulse per cycle	Cadence	Self-selected vs quick	-0.921	-1.054	0.133 [0.105, 0.160]	10.152 (19)	103.059 (1, 19)	4.126563e-09	4.126563e-09	0.844 [0.680, 0.910]
Right grip impulse per cycle	Cadence	Slow vs self-selected	-0.890	-1.031	0.141 [0.130, 0.153]	26.185 (19)	685.679 (1, 19)	2.261808e-16	4.523615e-16	0.973 [0.943, 0.984]
Right grip impulse per cycle	Cadence	Slow vs quick	-0.890	-1.173	0.283 [0.261, 0.305]	26.897 (19)	723.474 (1, 19)	1.376022e-16	4.128067e-16	0.974 [0.946, 0.985]
Right grip impulse per cycle	Cadence	Self-selected vs quick	-1.031	-1.173	0.142 [0.120, 0.163]	13.852 (19)	191.878 (1, 19)	2.214082e-11	2.214082e-11	0.910 [0.811, 0.948]
Left pedal peak force	Cadence	Slow vs self-selected	1.188	1.252	-0.064 [-0.081, -0.047]	-7.992 (15)	63.874 (1, 15)	8.716208e-07	1.743242e-06	0.810 [0.575, 0.896]
Left pedal peak force	Cadence	Slow vs quick	1.188	1.318	-0.130 [-0.153, -0.108]	-12.226 (15)	149.485 (1, 15)	3.346773e-09	1.004032e-08	0.909 [0.787, 0.950]
Left pedal peak force	Cadence	Self-selected vs quick	1.252	1.318	-0.066 [-0.094, -0.038]	-5.067 (15)	25.678 (1, 15)	0.000139	0.000139	0.631 [0.271, 0.797]
Left pedal impulse per cycle	Cadence	Slow vs self-selected	0.081	-0.031	0.113 [0.096, 0.129]	14.486 (14)	209.830 (1, 14)	8.069058e-10	2.420717e-09	0.937 [0.847, 0.967]
Left pedal impulse per cycle	Cadence	Slow vs quick	0.081	-0.127	0.209 [0.174, 0.244]	12.842 (14)	164.913 (1, 14)	3.898953e-09	7.797906e-09	0.922 [0.810, 0.958]
Left pedal impulse per cycle	Cadence	Self-selected vs quick	-0.031	-0.127	0.096 [0.059, 0.133]	5.532 (14)	30.608 (1, 14)	7.382494e-05	7.382494e-05	0.686 [0.336, 0.831]
Right pedal peak force	Cadence	Slow vs self-selected	1.177	1.257	-0.081 [-0.103, -0.058]	-7.609 (15)	57.901 (1, 15)	1.586605e-06	4.259453e-06	0.794 [0.544, 0.888]
Right pedal peak force	Cadence	Slow vs quick	1.177	1.290	-0.114 [-0.145, -0.082]	-7.679 (15)	58.972 (1, 15)	1.419818e-06	4.259453e-06	0.797 [0.550, 0.890]
Right pedal peak force	Cadence	Self-selected vs quick	1.257	1.290	-0.033 [-0.051, -0.015]	-3.858 (15)	14.883 (1, 15)	0.001549	0.001549	0.498 [0.119, 0.720]
Right pedal impulse per cycle	Cadence	Slow vs self-selected	0.080	-0.020	0.099 [0.072, 0.127]	7.798 (14)	60.813 (1, 14)	1.841241e-06	1.841241e-06	0.813 [0.568, 0.900]
Right pedal impulse per cycle	Cadence	Slow vs quick	0.080	-0.127	0.206 [0.171, 0.241]	12.612 (14)	159.054 (1, 14)	4.928415e-09	1.478524e-08	0.919 [0.803, 0.957]
Right pedal impulse per cycle	Cadence	Self-selected vs quick	-0.020	-0.127	0.107 [0.086, 0.128]	10.840 (14)	117.496 (1, 14)	3.411138e-08	6.822276e-08	0.894 [0.744, 0.943]
Saddle impulse per cycle	Cadence	Slow vs self-selected	0.527	0.380	0.147 [0.117, 0.177]	10.139 (19)	102.809 (1, 19)	4.208680e-09	8.417361e-09	0.844 [0.680, 0.910]
Saddle impulse per cycle	Cadence	Slow vs quick	0.527	0.259	0.268 [0.223, 0.313]	12.567 (19)	157.924 (1, 19)	1.183697e-10	3.551091e-10	0.893 [0.776, 0.938]
Saddle impulse per cycle	Cadence	Self-selected vs quick	0.380	0.259	0.121 [0.078, 0.164]	5.854 (19)	34.270 (1, 19)	1.225559e-05	1.225559e-05	0.643 [0.339, 0.792]
Left grip cycle-averaged vertical force	Posture	Comfort vs neutral	-0.752	-0.671	-0.082 [-0.115, -0.049]	-5.220 (19)	27.246 (1, 19)	4.877650e-05	0.000146	0.589 [0.265, 0.759]
Left grip cycle-averaged vertical force	Posture	Comfort vs athletic	-0.752	-0.692	-0.061 [-0.098, -0.024]	-3.472 (19)	12.053 (1, 19)	0.002554	0.005108	0.388 [0.069, 0.630]
Left grip cycle-averaged vertical force	Posture	Neutral vs athletic	-0.671	-0.692	0.021 [-0.009, 0.051]	1.462 (19)	2.139 (1, 19)	0.159975	0.159975	0.101 [0.000, 0.386]
Right grip cycle-averaged vertical force	Posture	Comfort vs neutral	-0.857	-0.797	-0.060 [-0.090, -0.030]	-4.238 (19)	17.958 (1, 19)	0.000445	0.001336	0.486 [0.149, 0.695]
Right grip cycle-averaged vertical force	Posture	Comfort vs athletic	-0.857	-0.796	-0.061 [-0.092, -0.030]	-4.107 (19)	16.865 (1, 19)	0.000600	0.001336	0.470 [0.135, 0.684]
Right grip cycle-averaged vertical force	Posture	Neutral vs athletic	-0.797	-0.796	-0.001 [-0.042, 0.041]	-0.032 (19)	0.001 (1, 19)	0.974428	0.974428	0.000 [0.000, 0.002]
Left pedal cycle-averaged vertical force	Cadence	Slow vs self-selected	0.142	0.177	-0.035 [-0.053, -0.017]	-4.195 (14)	17.599 (1, 14)	0.000899	0.001798	0.557 [0.164, 0.759]
Left pedal cycle-averaged vertical force	Cadence	Slow vs quick	0.142	0.209	-0.067 [-0.091, -0.042]	-5.861 (14)	34.353 (1, 14)	4.137700e-05	0.000124	0.710 [0.376, 0.845]
Left pedal cycle-averaged vertical force	Cadence	Self-selected vs quick	0.177	0.209	-0.032 [-0.065, 0.001]	-2.073 (14)	4.296 (1, 14)	0.057149	0.057149	0.235 [0.000, 0.551]
Right pedal cycle-averaged vertical force	Cadence	Slow vs self-selected	0.140	0.189	-0.048 [-0.066, -0.030]	-5.669 (14)	32.133 (1, 14)	5.798120e-05	0.000156	0.697 [0.353, 0.837]
Right pedal cycle-averaged vertical force	Cadence	Slow vs quick	0.140	0.210	-0.069 [-0.095, -0.043]	-5.730 (14)	32.836 (1, 14)	5.201080e-05	0.000156	0.701 [0.361, 0.839]

Outcome	Main effect	Comparison	Mean 1	Mean 2	Mean difference [95% CI]	t (df)	F (df1, df2)	praw	pHolm	ηp² [95% CI]
Right pedal cycle-averaged vertical force	Cadence	Self-selected vs quick	0.189	0.210	-0.021 [-0.044, 0.001]	-2.005 (14)	4.019 (1, 14)	0.064726	0.064726	0.223 [0.000, 0.542]

Table S13. Simple-effects comparisons for significant cadence × gear ratio interactions after natural-log transformation (sensitivity analysis).

Outcome	Simple effect	Condition	Comparison	Mean 1	Mean 2	Mean difference [95% CI]	t (df)	F (df1, df2)	praw	pHolm	ηp² [95% CI]
Right grip impulse per cycle	Cadence within gear ratio	Light	Slow vs self-selected	-0.839	-0.945	0.106 [0.081, 0.130]	9.106 (19)	82.921 (1, 19)	2.326337e-08	4.652674e-08	0.814 [0.622, 0.892]
Right grip impulse per cycle	Cadence within gear ratio	Light	Slow vs quick	-0.839	-1.075	0.236 [0.213, 0.258]	21.917 (19)	480.342 (1, 19)	5.995691e-15	3.597415e-14	0.962 [0.919, 0.978]
Right grip impulse per cycle	Cadence within gear ratio	Light	Self-selected vs quick	-0.945	-1.075	0.130 [0.110, 0.150]	13.571 (19)	184.165 (1, 19)	3.159727e-11	9.479180e-11	0.906 [0.804, 0.946]
Right grip impulse per cycle	Cadence within gear ratio	Heavy	Slow vs self-selected	-0.941	-1.118	0.177 [0.158, 0.196]	19.414 (19)	376.901 (1, 19)	5.464919e-14	2.732460e-13	0.952 [0.898, 0.972]
Right grip impulse per cycle	Cadence within gear ratio	Heavy	Slow vs quick	-0.941	-1.271	0.330 [0.287, 0.373]	15.961 (19)	254.755 (1, 19)	1.837210e-12	7.348840e-12	0.931 [0.854, 0.960]
Right grip impulse per cycle	Cadence within gear ratio	Heavy	Self-selected vs quick	-1.118	-1.271	0.153 [0.118, 0.189]	9.025 (19)	81.454 (1, 19)	2.673954e-08	4.652674e-08	0.811 [0.617, 0.891]
Right grip impulse per cycle	Gear ratio within cadence	Slow	Heavy vs light	-0.941	-0.839	-0.102 [-0.150, -0.053]	-4.404 (19)	19.392 (1, 19)	0.000305	0.000305	0.505 [0.168, 0.707]
Right grip impulse per cycle	Gear ratio within cadence	Self-selected	Heavy vs light	-1.118	-0.945	-0.173 [-0.212, -0.134]	-9.262 (19)	85.793 (1, 19)	1.781694e-08	5.345081e-08	0.819 [0.631, 0.895]
Right grip impulse per cycle	Gear ratio within cadence	Quick	Heavy vs light	-1.271	-1.075	-0.196 [-0.248, -0.144]	-7.888 (19)	62.217 (1, 19)	2.065981e-07	4.131962e-07	0.766 [0.535, 0.865]
Right grip cycle-averaged vertical force	Cadence within gear ratio	Light	Slow vs self-selected	-0.763	-0.724	-0.040 [-0.064, -0.015]	-3.381 (19)	11.432 (1, 19)	0.003135	0.012542	0.376 [0.061, 0.621]
Right grip cycle-averaged vertical force	Cadence within gear ratio	Light	Slow vs quick	-0.763	-0.727	-0.036 [-0.063, -0.009]	-2.788 (19)	7.773 (1, 19)	0.011727	0.035181	0.290 [0.018, 0.559]
Right grip cycle-averaged vertical force	Cadence within gear ratio	Light	Self-selected vs quick	-0.724	-0.727	0.004 [-0.018, 0.026]	0.348 (19)	0.121 (1, 19)	0.732020	0.732020	0.006 [0.000, 0.207]
Right grip cycle-averaged vertical force	Cadence within gear ratio	Heavy	Slow vs self-selected	-0.865	-0.897	0.032 [0.013, 0.050]	3.622 (19)	13.122 (1, 19)	0.001813	0.010880	0.409 [0.084, 0.643]
Right grip cycle-averaged vertical force	Cadence within gear ratio	Heavy	Slow vs quick	-0.865	-0.924	0.058 [0.024, 0.093]	3.525 (19)	12.428 (1, 19)	0.002262	0.011308	0.395 [0.074, 0.635]

Outcome	Simple effect	Condition	Comparison	Mean 1	Mean 2	Mean difference [95% CI]	t (df)	F (df1, df2)	praw	pHolm	ηp² [95% CI]
Right grip cycle-averaged vertical force	Cadence within gear ratio	Heavy	Self-selected vs quick	-0.897	-0.924	0.027 [-0.005, 0.058]	1.767 (19)	3.124 (1, 19)	0.093221	0.186442	0.141 [0.000, 0.430]
Right grip cycle-averaged vertical force	Gear ratio within cadence	Slow	Heavy vs light	-0.865	-0.763	-0.102 [-0.150, -0.053]	-4.404 (19)	19.392 (1, 19)	0.000305	0.000305	0.505 [0.168, 0.707]
Right grip cycle-averaged vertical force	Gear ratio within cadence	Self-selected	Heavy vs light	-0.897	-0.724	-0.173 [-0.212, -0.134]	-9.262 (19)	85.793 (1, 19)	1.781690e-08	5.345080e-08	0.819 [0.631, 0.895]
Right grip cycle-averaged vertical force	Gear ratio within cadence	Quick	Heavy vs light	-0.924	-0.727	-0.196 [-0.248, -0.144]	-7.888 (19)	62.217 (1, 19)	2.065980e-07	4.131960e-07	0.766 [0.535, 0.865]

Note: Holm correction was applied within each follow-up family. The left- and right-pedal impulse cadence × gear ratio interactions were nonsignificant after log transformation and therefore were not followed up on the transformed scale.
